# Evaluation of penalized and machine learning methods for asthma disease prediction in the Korean Genome and Epidemiology Study (KoGES)

**DOI:** 10.1186/s12859-024-05677-x

**Published:** 2024-02-02

**Authors:** Yongjun Choi, Junho Cha, Sungkyoung Choi

**Affiliations:** 1 Department of Applied Artificial Intelligence, College of Computing, Hanyang University, 55 Hanyang-daehak-ro, Sangnok-gu, Ansan, 15588 South Korea; 2 Department of Mathematical Data Science, College of Science and Convergence Technology, Hanyang University, 55 Hanyang-daehak-ro, Sangnok-gu, Ansan, 15588 South Korea

**Keywords:** Disease risk prediction model, Large-scale genetic data, Asthma, Penalized methods, Machine learning methods, Ensemble methods, Genome-wide association study, GWAS, Korean Genome and Epidemiology Study, KoGES, Oversampling

## Abstract

**Background:**

Genome-wide association studies have successfully identified genetic variants associated with human disease. Various statistical approaches based on penalized and machine learning methods have recently been proposed for disease prediction. In this study, we evaluated the performance of several such methods for predicting asthma using the Korean Chip (KORV1.1) from the Korean Genome and Epidemiology Study (KoGES).

**Results:**

First, single-nucleotide polymorphisms were selected via single-variant tests using logistic regression with the adjustment of several epidemiological factors. Next, we evaluated the following methods for disease prediction: ridge, least absolute shrinkage and selection operator, elastic net, smoothly clipped absolute deviation, support vector machine, random forest, boosting, bagging, naïve Bayes, and *k*-nearest neighbor. Finally, we compared their predictive performance based on the area under the curve of the receiver operating characteristic curves, precision, recall, F1-score, Cohen′s Kappa, balanced accuracy, error rate, Matthews correlation coefficient, and area under the precision-recall curve. Additionally, three oversampling algorithms are used to deal with imbalance problems.

**Conclusions:**

Our results show that penalized methods exhibit better predictive performance for asthma than that achieved via machine learning methods. On the other hand, in the oversampling study, randomforest and boosting methods overall showed better prediction performance than penalized methods.

**Supplementary Information:**

The online version contains supplementary material available at 10.1186/s12859-024-05677-x.

## Background

Asthma is a major global health problem estimated to affect approximately 334 million people in 2019 [1–3]. It is a leading cause of rhinitis, chronic bronchitis, heart disease, stroke, vascular disease, arthritis, and osteoporosis [4]. Although there is no cure for asthma, several medications can be used to treat and control the symptoms. However, the treatment of early-onset asthma patients results in a considerable socioeconomic burden due to substantial medical expenses and decreased work productivity of the affected individuals [2, 5].

Over the last decade, genome-wide association studies (GWASs) have identified 140 susceptibility single-nucleotide polymorphisms (SNPs) associated with asthma, greatly advancing our understanding of asthma genetics [6–12]. Thus, potentially causal SNPs in genes or gene sets also contribute to the construction of more informed prediction models. Despite these advances, discovered asthma-associated loci explain only a small fraction of overall disease heritability [6, 8, 13]. In fact, most complex disease susceptibility loci identified via GWASs have rather modest effects, except for in the case of Mendelian dyslipidemias [14]. The huge number of genetic variants identified from a small number of samples (or so-called “large P and small N” problem) [15] represents a major challenge in predictive model construction. Regression analysis does not account for the multicollinearity caused by linkage disequilibrium among predictor SNPs and can therefore yield misleading results [16]. Many penalized and machine learning methods have recently been proposed to solve these issues. However, a comprehensive evaluation of the existing approaches for disease risk prediction has not yet been conducted.

The most popular approach for constructing a disease risk prediction model employs a simple linear (logistic) regression model with genotype scores [17–19]. Regression coefficients of previously known disease-associated SNPs are estimated using a training dataset. The sum of regression coefficients for each individual can then be incorporated to construct the disease risk prediction model for the test dataset. Many studies have shown that a genetic score-based approach for predicting disease risk is partially helpful [20]. However, these approaches often show reduced predictive performance for complex diseases [21, 22]. Understanding the causes of complex diseases, such as cancer, diabetes, and asthma, can be improved by considering complex genetic and environmental risk factors as well as gene–gene and gene-environment interactions.

As an alternative to the genetic score-based approach, machine learning algorithms have been widely used to improve disease risk prediction performance. For example, support vector machines (SVMs) [23] often outperform other classification methods in terms of classification accuracy [24]. Furthermore, several studies have shown that ensemble methods, particularly random forest (RF) [25], boosting [26], bagging [27], naïve Bayes (NB) [28], and *k*-nearest neighbor classification (KNN) [29], improve the prediction of complex diseases [30, 31]. However, despite advances in machine learning algorithms, certain limitations remain. Machine learning algorithms find it difficult to interpret the underlying genetic factors of disease in the prediction model. Furthermore, these approaches do not provide conditional probabilities for each individual prediction [32].

Penalized methods, such as ridge [33–35], least absolute shrinkage and selection operator (Lasso) [36], elastic net (Enet) [37], and smoothly clipped absolute deviation (SCAD), have been proposed to solve large P and small N problems [38]. Although penalized methods yield biased estimates by considering the regression coefficients as zero, these regression coefficient estimates will have a small variance. Thus, such approaches enhance the accuracy of predictions because of their small mean squared error [39]. In addition, many penalized methods have recently been used for variable selection in large-scale genetic data [40–44].

In this study, we compared the performance of various penalized and machine learning methods for predicting asthma development using data from the Korean Genome and Epidemiology Study (KoGES) [45–47]. We considered the following methods for disease risk prediction: ridge, Lasso, Enet, SCAD, SVM, RF, boosting, bagging, NB, and KNN. The predictive performances of penalized and machine learning methods were compared using the area under the curve (AUC) of the receiver operating characteristic (ROC) curves, precision, recall, F1-score, Cohen′s Kappa, balanced accuracy (BA), error rate, Matthews correlation coefficient (MCC), and area under the precision-recall curve (AUPRC), which are the most widely used methods for evaluating prediction performance.

For an imbalanced dataset, most prediction methods are not able to establish meaningful classifiers. Therefore, many approaches have been proposed to address the class imbalance, in which the most commonly used technique is oversampling or undersampling algorithms. The oversampling algorithms generate the synthetic data points belonging to the minority class to obtain the desired balancing ratio. In contrast, the undersampling algorithms remove several data points from the majority class. In this study, we utilize several oversampling algorithms for handling an imbalanced dataset, including the majority weighted minority oversampling technique (MWMOTE) [48], the random walk oversampling (RWO) [49], and the synthetic minority oversampling technique (SMOTE) [50]. The most famous oversampling algorithm is SMOTE, which generates synthetic data from the minor class using KNN. The MWMOTE is an extension of the SMOTE algorithm that assigns a higher weight to borderline samples, minority clusters and examples near the borderline of the two classes. The RWO algorithm, motivated by the central limit theorem, generates synthetic samples so that the mean and deviation of numerical attributes remain as close as the original ones.

Finally, We inferred the pathogenicity and deleteriousness of the observed variants via combined annotation-dependent depletion (CADD) [51] and deleterious annotation of genetic variants using neural network (DANN) scores [52], which take genetic, evolutionary, structural, functional, and biochemical properties into account.

## Results

### Demographic characteristics

Table 1 shows the distribution of demographic characteristics of unaffected participants (controls) and patients with asthma (cases) in each cohort of the KoGES. Of the 3,003 participants in the Cardiovascular Disease Association Study (CAVAS) cohort, 2,908 (96.8%) were controls, and 95 (3.2%) were patients. Among the 5,420 participants in the Korea Association Resource Study (KARE) cohort, 5,308 (97.9%) were controls, and 112 (2.1%) were patients. Of the 58,434 participants in the Health Examinees Study (HEXA) cohort, 57,459 (98.3%) were controls, and 975 (1.7%) were patients. The associations of asthma with environmental risk factors (smoking status and allergy status) and human anthropometric dimensions (sex, age, and body mass index [BMI]) were analyzed in the CAVAS, KARE, and HEXA cohorts using the *t*-test and chi-square test, respectively (Table 1). The demographic analysis demonstrated that asthma was significantly associated with age (*p* = 0.0025 in the CAVAS cohort, *p* = 0.0026 in the KARE cohort, *p* < 0.0001 in the HEXA cohort) and allergy status (*p* < 0.0001 in all cohorts). As shown in Table 1, asthma was significantly associated with sex (*p* = 0.0048 in the KARE cohort and *p* < 0.0001 in the HEXA cohort) and BMI (*p* = 0.0002 in the CAVAS and HEXA cohorts). Although smoking status was not associated with asthma in this study, it was considered a covariate in many previous studies on asthma prediction [53–55].

**Table 1:** Demographic variables for the CAVAS, KARE, and HEXA cohorts

	CAVAS		*p*^a^	KARE		*p*	HEXA		*p*
	Case (*n* = 95)	Control (*n* = 2908)		Case (*n* = 112)	Control (*n* = 5308)		Case (*n* = 975)	Control (*n* = 57,459)	
Sex									
Male	37 (3.1%)	1164 (96.9%)	0.8325	39 (1.5%)	2563 (98.5%)	0.0048	283 (1.4%)	19,924 (98.6%)	< 0.0001
Female	58 (3.2%)	1744 (96.8%)		73 (2.6%)	2745 (97.4%)		692 (1.8%)	37,535 (98.2%)	
Age (years)^b^	57.9 ± 7.8	55.4 ± 7.8	0.0025	53.3 ± 7.9	51.5 ± 8.5	0.0026	55.4 ± 8.4	53.8 ± 8.0	< 0.0001
BMI (kg/m^2^)	25.5 ± 3.4	24.5 ± 3.0	0.0002	25.0 ± 3.5	24.6 ± 3.0	0.1536	24.3 ± 3.2	23.9 ± 2.9	0.0002
Smoking status^c^									
Non-Smokers	72 (3.0%)	2123 (97.0%)	0.5471	71 (2.2%)	3173 (97.8%)	0.4397	721 (1.7%)	42,070 (98.3%)	0.6348
Smokers	23 (2.8%)	785 (97.2%)		41 (1.9%)	2135 (98.1%)		254 (1.6%)	15,389 (98.4%)	
Allergy status									
Non-allergy	74 (2.7%)	2695 (97.3%)	< 0.0001	86 (1.7%)	5015 (98.3%)	< 0.0001	727 (1.3%)	53,642 (98.7%)	< 0.0001
Allergy	21 (9.0%)	213 (91.0%)		26 (8.2%)	293 (91.8%)		248 (6.1%)	3,817 (93.9%)	

^a^*p*-value from *t*-test or chi-square test

^b^Means ± standard deviation (SD)

^c^Smoking status (No: never smoker, Yes: former smoker or current smoker)

### Comparison of the predictive performance

To compare the performance of the penalized and machine learning methods, we calculated the AUCs of those methods on the test dataset using the R-package *pROC* [56]. Their performances were also assessed based on precision, recall, F1-score, Cohen′s Kappa, BA, and error rate using the *caret* package in R [57]. The MCC and AUPRC were performed using the R-package *mltools* and *precrec* packages, respectively [58, 59]. Table 2 and Additional file 1: Tables S1-S2 illustrate that the relative performance of each method generally depended on the number of SNPs within the cohorts. These performance measurements may be explained by the relative importance of genetic components in asthma. We calculated the proportion of variances, *h*^2^, for asthma explained by the top SNP sets (50, 100, 200, and 400 SNPs) and SNP-based heritability [60] using the genomic relatedness-based restricted maximum-likelihood approaches implemented in the GCTA program [61]. As shown in Table 3, the heritability estimates for asthma ranged from 16.6% to 45.7% in the CAVAS cohort, 7.4% to 29.0% in the KARE cohort, and 0.7% to 4.9% in the HEXA cohort. Our findings reveal that various evaluation metrics showed higher values in the CAVAS cohort compared to those observed in the KARE and HEXA cohorts.

**Table 2:** Performance evaluation metrics for prediction methods in the CAVAS cohort using the test dataset

# of SNPs	Metrics	Ridge	Lasso	Enet	SCAD	SVM	RF	Boosting	Bagging	NB	KNN
50	AUC	0.795	0.805	0.802	0.794	0.659	0.777	0.748	0.618	0.769	0.692
	Precision	0.183	0.146	0.148	0.120	0.065	0.091	0.147	0.060	0.117	0.032
	Recall	0.684	0.632	0.632	0.684	0.684	0.684	0.579	0.421	0.737	1.000
	F1-score	0.289	0.238	0.240	0.205	0.118	0.160	0.234	0.105	0.201	0.061
	Cohen′s Kappa	0.251	0.196	0.199	0.159	0.064	0.111	0.193	0.053	0.155	0.000
	Balanced accuracy	0.792	0.756	0.756	0.760	0.680	0.730	0.734	0.603	0.777	0.500
	Error rate	0.107	0.128	0.127	0.168	0.323	0.227	0.120	0.227	0.185	0.968
	MCC	0.317	0.261	0.263	0.237	0.134	0.189	0.248	0.087	0.243	0.000
	AUPRC	0.204	0.303	0.303	0.296	0.192	0.156	0.118	0.064	0.106	0.088
100	AUC	0.893	0.890	0.890	0.889	0.856	0.822	0.817	0.792	0.808	0.584
	Precision	0.115	0.214	0.214	0.221	0.169	0.090	0.086	0.032	0.060	0.138
	Recall	0.895	0.789	0.789	0.789	0.737	0.737	0.842	1.000	0.947	0.211
	F1-score	0.204	0.337	0.337	0.345	0.275	0.161	0.157	0.061	0.114	0.167
	Cohen′s Kappa	0.156	0.302	0.302	0.311	0.235	0.111	0.105	0.000	0.057	0.134
	Balanced accuracy	0.835	0.847	0.847	0.849	0.809	0.747	0.776	0.500	0.733	0.584
	Error rate	0.222	0.098	0.098	0.095	0.123	0.243	0.287	0.968	0.468	0.067
	MCC	0.272	0.379	0.379	0.386	0.313	0.198	0.209	0.000	0.163	0.137
	AUPRC	0.382	0.391	0.393	0.397	0.389	0.307	0.171	0.131	0.105	0.090
200	AUC	0.949	0.947	0.954	0.946	0.930	0.832	0.810	0.823	0.809	0.610
	Precision	0.187	0.224	0.273	0.236	0.107	0.092	0.140	0.116	0.140	0.032
	Recall	0.895	0.895	0.947	0.895	1.000	0.789	0.789	0.737	0.632	1.000
	F1-score	0.309	0.358	0.424	0.374	0.193	0.165	0.238	0.200	0.229	0.061
	Cohen′s Kappa	0.271	0.324	0.394	0.341	0.144	0.115	0.195	0.154	0.186	0.000
	Balanced accuracy	0.884	0.897	0.932	0.900	0.863	0.767	0.816	0.776	0.752	0.500
	Error rate	0.127	0.102	0.082	0.095	0.265	0.253	0.160	0.187	0.135	0.968
	MCC	0.375	0.418	0.484	0.431	0.278	0.211	0.289	0.241	0.252	0.000
	AUPRC	0.572	0.557	0.548	0.559	0.483	0.219	0.255	0.200	0.152	0.134
400	AUC	0.977	0.986	0.983	0.928	0.985	0.896	0.889	0.809	0.883	0.593
	Precision	0.548	0.429	0.346	0.200	0.279	0.158	0.158	0.124	0.106	0.032
	Recall	0.895	0.947	0.947	0.895	1.000	0.789	0.789	0.684	0.895	1.000
	F1-score	0.680	0.590	0.507	0.327	0.437	0.263	0.263	0.210	0.189	0.061
	Cohen′s Kappa	0.667	0.571	0.483	0.290	0.407	0.222	0.222	0.165	0.140	0.000
	Balanced accuracy	0.935	0.953	0.944	0.889	0.958	0.826	0.826	0.763	0.823	0.500
	Error rate	0.027	0.042	0.058	0.117	0.082	0.140	0.140	0.163	0.243	0.968
	MCC	0.689	0.622	0.553	0.391	0.506	0.313	0.313	0.242	0.256	0.000
	AUPRC	0.802	0.825	0.815	0.410	0.776	0.259	0.346	0.222	0.162	0.137

**Table 3:** Proportion of variance explained by genotyped single-nucleotide polymorphisms

Cohort		50 SNPs	100 SNPs	200 SNPs	400 SNPs
CAVAS	*h*^2^	0.166	0.260	0.359	0.457
	*σ*(*h*^2^)	0.069	0.059	0.052	0.040
KARE	*h*^2^	0.074	0.119	0.189	0.290
	*σ*(*h*^2^)	0.022	0.026	0.028	0.030
HEXA	*h*^2^	0.007	0.011	0.030	0.049
	*σ*(*h*^2^)	0.002	0.003	0.006	0.006

Table 2 and Additional file 1: Tables S1–S2 show that evaluation metrics such as precision, F1-score, Cohen′s Kappa, and MCC produced low results that indicated a problem in evaluating the prediction models. Furthermore, recall for the SVM and KNN methods also produced low scores that correctly reflected the prediction issue. In the case of an imbalanced dataset, the predictive methods were not able to correctly recognize positive data instances and therefore produced a confusion matrix with a low number of true positives (i.e., case-patient ratio: 3.2% in the CAVAS cohort, 2.1% in the KARE cohort, and 1.7% in the HEXA cohort). Therefore, the results were mainly examined by focusing on AUC and AUPRC as an indicator of model performance.

**Fig. 1:**
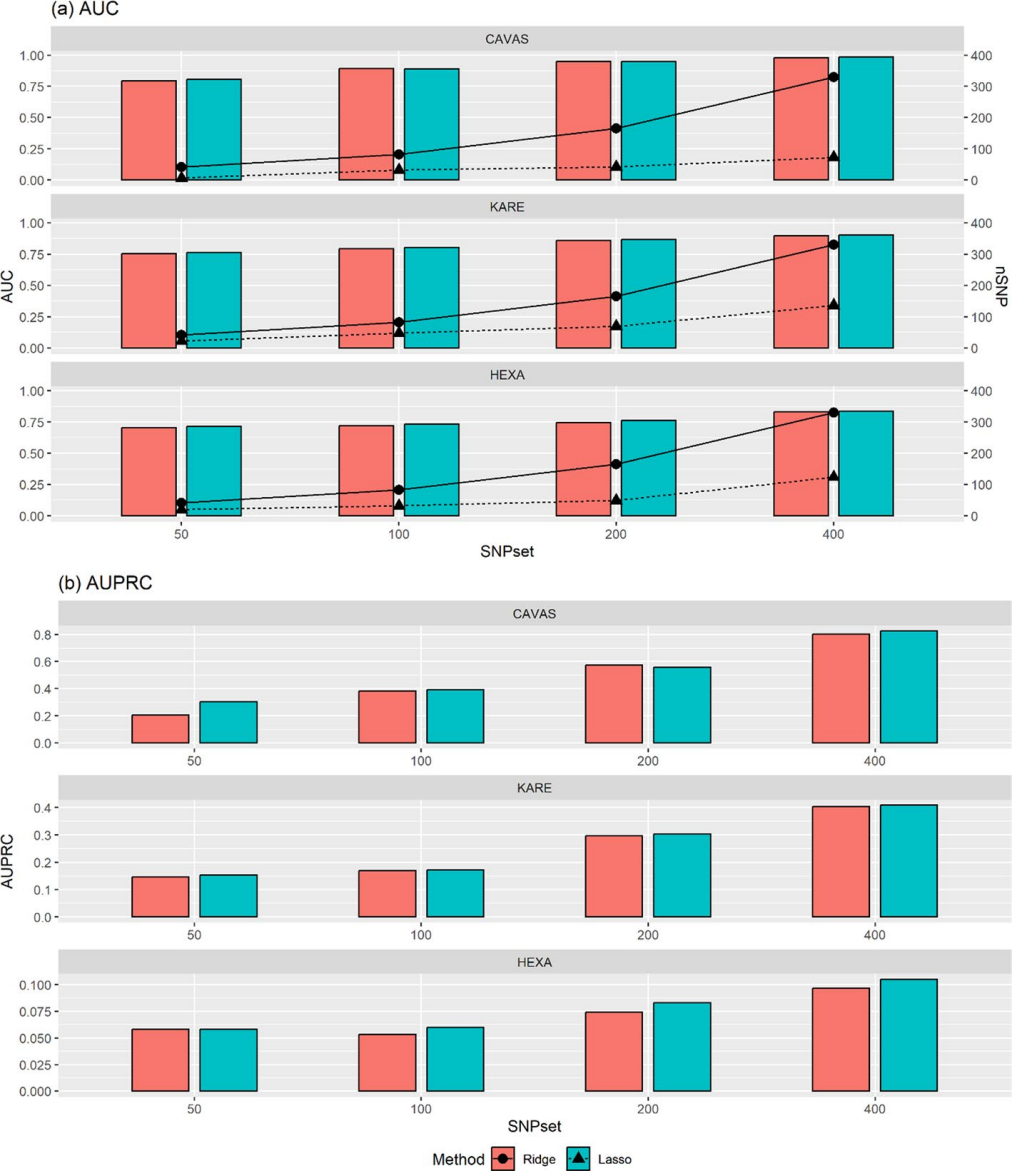
Comparison of **a** AUCs and non-zero estimate parameters of SNPs, and **b** AUPRCs for ridge and Lasso predictions using test datasets. CAVAS, Cardiovascular Disease Association Study; KARE, Korea Association Resource Study; HEXA, Health Examinees Study; AUC, area under the curve; AUPRC, area under the precision-recall curve; SNP, single-nucleotide polymorphism; Lasso, least absolute shrinkage and selection operator

The AUCs of penalized methods on the test sets were outperformed by the machine learning methods across the various top SNP sets in Table 2 and Additional file 1: Tables S1–S2. Some of the differences in performance may be explained by penalized methods shrinking the estimated causal SNP weights, which is useful for reducing the effects of overfitting. Overall performance comparison among penalized methods shows that Lasso and Enet exhibited the best performance, followed by the ridge and then SCAD, even though the performance of each penalized method depends on the cohort and the size of top SNP sets. The robustness of Lasso with a variable selection could be explained by lower model complexity when compared to the ridge method in Fig. 1a. For instance, Lasso usually selected a small number of classifiers (8 SNPs of the top 50 SNPs and 88 SNPs of the top 400 SNPs in the CAVAS cohort) but achieved similar or higher predictive accuracy than the ridge method. Therefore, Lasso was able to deal with a large number of SNPs for better risk estimation than non-penalized or ridge methods. Comparing the overall performances of machine learning methods, it can be seen that SVM exhibited the best performance; RF, Boosting, and NB exhibited the second best performance, followed by bagging and, finally, KNN. For instance, utilizing the top 50 SNPs in the CAVAS cohort, Lasso established the best model, with an AUC of 0.805, while SVM had an AUC of 0.659.

In Table 2 and Additional file 1: Tables S1-S2, the improvement of AUPRC for the penalized methods with a larger number of SNPs is more significant than the machine learning methods. Figure 1b shows that the Lasso method achieved similar or higher AUPRCs than the ridge method across various scenarios. For example, the Lasso method consistently selected a relatively small number of SNPs (at most 8 SNPs for the top 50 SNPs set and 88 SNPs for the top 400 SNPs sets) but achieved higher AUPRC values than the ridge method when con-sidering the top 400 SNP sets in CAVAS cohort. Thus, we can conclude that the Lasso method seems a more reasonable choice than the Ridge method in terms of model complexity.

### Comparison of the prediction performance of methods with oversampling algorithms

To handle the imbalance problem, we analyzed the prediction model's performance using three oversampling techniques, including MWMOTE, RWO, and SMOTE, which were performed using the R-package *imbalance* [62]. The oversampling algorithms were applied only to the training set during a cross-validation (CV) procedure. The penalized and machine learning methods were built, tuned, and trained on an oversampled training set with five-fold CVs, followed by validation on the testing set across the top SNP sets (50, 100, and 200 SNPs) in all cohorts.

Table 4 and Additional file 1: Tables S3–S4 show the various additional evaluation metrics of predictive methods with oversampling algorithms. Similar to the original data set analysis results, some evaluation metrics such as precision, F1-score, Cohen′s Kappa, and MCC still showed low results. Furthermore, recall and error rate measures showed wide variability depending on oversampling algorithms. Therefore, we focused on the AUC and AUPRC evaluation as a key indicator of model performance.

**Table 4:** Comparison of the prediction performance of methods with oversampling algorithms in the CAVAS cohort using the test dataset

Algorithm	# of SNP	Metrics	Ridge	Lasso	Enet	SCAD	SVM	RF	Boosting	Bagging	NB	KNN
MWMOTE	50	AUC	0.653	0.641	0.639	0.642	0.552	0.652	0.624	0.715	0.484	0.622
		Precision	0.109	0.049	0.048	0.051	0.010	0.045	0.000	0.084	0.022	0.042
		Recall	0.438	0.733	0.800	0.733	0.062	1.000	0.000	0.533	0.533	0.938
		F1-score	0.175	0.092	0.090	0.096	0.017	0.086	NaN	0.145	0.043	0.080
		Cohen′s Kappa	0.129	0.036	0.033	0.040	− 0.043	0.028	− 0.048	0.097	− 0.018	0.018
		Balanced accuracy	0.658	0.639	0.643	0.648	0.424	0.656	0.456	0.673	0.392	0.600
		Error rate	0.137	0.450	0.504	0.431	0.238	0.667	0.117	0.196	0.740	0.714
		MCC	0.167	0.097	0.100	0.104	− 0.067	0.118	− 0.055	0.151	− 0.085	0.082
		AUPRC	0.065	0.110	0.112	0.063	0.034	0.044	0.038	0.057	0.045	0.042
	100	AUC	0.669	0.685	0.706	0.681	0.561	0.698	0.784	0.772	0.368	0.622
		Precision	0.212	0.066	0.066	0.055	0.068	0.053	0.096	0.063	0.000	0.042
		Recall	0.467	0.733	0.733	0.867	0.333	0.800	0.733	0.800	0.000	0.733
		F1-score	0.292	0.122	0.121	0.103	0.114	0.100	0.171	0.118	NaN	0.080
		Cohen′s Kappa	0.260	0.068	0.067	0.047	0.065	0.044	0.122	0.063	− 0.059	0.023
		Balanced accuracy	0.705	0.700	0.699	0.691	0.594	0.671	0.756	0.710	0.370	0.600
		Error rate	0.071	0.331	0.333	0.473	0.162	0.450	0.223	0.375	0.283	0.525
		MCC	0.282	0.146	0.145	0.133	0.091	0.119	0.209	0.149	− 0.104	0.070
		AUPRC	0.202	0.054	0.060	0.053	0.041	0.063	0.091	0.112	0.022	0.054
	200	AUC	0.788	0.748	0.756	0.740	0.610	0.704	0.731	0.725	0.430	0.560
		Precision	0.074	0.072	0.082	0.071	0.057	0.056	0.075	0.054	0.034	0.039
		Recall	0.933	0.867	0.800	0.867	0.600	0.867	0.688	0.933	0.800	0.533
		F1-score	0.137	0.133	0.148	0.131	0.105	0.106	0.135	0.102	0.065	0.072
		Cohen′s Kappa	0.083	0.080	0.097	0.078	0.050	0.050	0.080	0.045	0.005	0.015
		Balanced accuracy	0.777	0.753	0.755	0.751	0.641	0.699	0.698	0.702	0.531	0.554
		Error rate	0.369	0.354	0.288	0.358	0.321	0.458	0.292	0.515	0.721	0.427
		MCC	0.197	0.181	0.192	0.180	0.104	0.139	0.154	0.141	0.025	0.038
		AUPRC	0.080	0.099	0.085	0.094	0.040	0.080	0.085	0.061	0.025	0.040
RWO	50	AUC	0.715	0.717	0.734	0.717	0.594	0.733	0.776	0.715	0.733	0.555
		Precision	0.065	0.105	0.090	0.096	0.073	0.066	0.064	0.055	0.058	0.057
		Recall	1.000	0.533	0.533	0.533	0.400	0.733	1.000	0.800	0.800	0.333
		F1-score	0.123	0.176	0.154	0.163	0.124	0.122	0.120	0.102	0.109	0.098
		Cohen′s Kappa	0.064	0.130	0.106	0.116	0.075	0.068	0.065	0.046	0.053	0.047
		Balanced accuracy	0.755	0.694	0.680	0.686	0.618	0.700	0.762	0.676	0.691	0.578
		Error rate	0.474	0.156	0.183	0.171	0.177	0.331	0.460	0.440	0.410	0.192
		MCC	0.182	0.184	0.161	0.171	0.109	0.146	0.183	0.123	0.135	0.071
		AUPRC	0.067	0.110	0.119	0.100	0.049	0.147	0.091	0.073	0.082	0.038
	100	AUC	0.790	0.780	0.771	0.759	0.729	0.827	0.816	0.748	0.849	0.628
		Precision	0.182	0.175	0.231	0.137	0.058	0.105	0.104	0.066	0.118	0.031
		Recall	0.667	0.667	0.600	0.667	0.800	0.733	0.867	0.812	0.867	1.000
		F1-score	0.286	0.278	0.333	0.227	0.109	0.183	0.186	0.122	0.208	0.061
		Cohen′s Kappa	0.249	0.240	0.302	0.185	0.053	0.136	0.138	0.065	0.162	0.000
		Balanced accuracy	0.785	0.783	0.768	0.766	0.691	0.766	0.813	0.709	0.829	0.500
		Error rate	0.104	0.108	0.075	0.142	0.410	0.204	0.238	0.387	0.206	0.969
		MCC	0.311	0.304	0.341	0.257	0.135	0.224	0.248	0.152	0.272	0.000
		AUPRC	0.239	0.162	0.153	0.134	0.091	0.139	0.100	0.074	0.170	0.043
	200	AUC	0.809	0.826	0.832	0.814	0.867	0.870	0.857	0.823	0.775	0.634
		Precision	0.095	0.104	0.080	0.084	0.087	0.141	0.112	0.090	0.110	0.031
		Recall	0.800	0.867	0.933	0.867	1.000	0.867	0.933	0.800	0.733	1.000
		F1-score	0.170	0.186	0.147	0.154	0.160	0.243	0.200	0.162	0.191	0.061
		Cohen′s Kappa	0.121	0.138	0.095	0.103	0.109	0.200	0.153	0.112	0.145	0.000
		Balanced accuracy	0.777	0.813	0.794	0.782	0.831	0.848	0.847	0.770	0.771	0.500
		Error rate	0.244	0.238	0.338	0.298	0.327	0.169	0.233	0.258	0.194	0.969
		MCC	0.219	0.248	0.212	0.210	0.240	0.308	0.275	0.210	0.232	0.000
		AUPRC	0.236	0.273	0.257	0.281	0.155	0.139	0.288	0.141	0.109	0.049
SMOTE	50	AUC	0.786	0.702	0.737	0.689	0.572	0.697	0.619	0.618	0.694	0.600
		Precision	0.067	0.056	0.071	0.050	0.047	0.063	0.056	0.049	0.056	0.056
		Recall	0.933	0.800	0.733	0.867	0.667	0.733	0.533	0.867	0.867	0.625
		F1-score	0.126	0.104	0.130	0.095	0.088	0.116	0.101	0.093	0.104	0.103
		Cohen′s Kappa	0.071	0.048	0.078	0.038	0.032	0.062	0.047	0.036	0.049	0.045
		Balanced accuracy	0.758	0.681	0.713	0.669	0.617	0.690	0.620	0.663	0.696	0.633
		Error rate	0.406	0.431	0.306	0.517	0.429	0.350	0.298	0.527	0.465	0.360
		MCC	0.181	0.126	0.159	0.118	0.082	0.138	0.091	0.114	0.136	0.098
		AUPRC	0.153	0.111	0.070	0.065	0.038	0.053	0.046	0.103	0.056	0.044
	100	AUC	0.834	0.751	0.764	0.750	0.563	0.812	0.795	0.690	0.545	0.625
		Precision	0.238	0.086	0.091	0.083	0.043	0.073	0.068	0.055	0.091	0.060
		Recall	0.667	0.800	0.800	0.800	0.600	0.867	1.000	0.875	0.267	0.467
		F1-score	0.351	0.156	0.163	0.150	0.080	0.135	0.127	0.104	0.136	0.106
		Cohen′s Kappa	0.320	0.105	0.114	0.099	0.023	0.082	0.073	0.045	0.093	0.054
		Balanced accuracy	0.799	0.763	0.771	0.757	0.585	0.756	0.778	0.682	0.590	0.615
		Error rate	0.077	0.271	0.256	0.283	0.429	0.348	0.429	0.499	0.106	0.246
		MCC	0.368	0.202	0.211	0.195	0.060	0.184	0.194	0.130	0.109	0.093
		AUPRC	0.298	0.099	0.109	0.091	0.054	0.092	0.085	0.124	0.043	0.054
	200	AUC	0.797	0.770	0.780	0.761	0.606	0.787	0.780	0.758	0.546	0.641
		Precision	0.080	0.069	0.078	0.065	0.043	0.066	0.082	0.065	0.051	0.055
		Recall	0.867	0.933	0.933	0.933	1.000	0.867	0.867	0.800	0.400	0.667
		F1-score	0.146	0.128	0.144	0.122	0.083	0.123	0.149	0.120	0.091	0.102
		Cohen′s Kappa	0.094	0.074	0.092	0.068	0.021	0.069	0.098	0.066	0.038	0.046
		Balanced accuracy	0.772	0.762	0.789	0.752	0.621	0.735	0.776	0.714	0.581	0.648
		Error rate	0.317	0.398	0.346	0.419	0.733	0.387	0.308	0.367	0.250	0.369
		MCC	0.200	0.185	0.208	0.176	0.102	0.167	0.204	0.153	0.065	0.106
		AUPRC	0.111	0.103	0.109	0.092	0.049	0.105	0.078	0.079	0.037	0.047

**Fig. 2:**
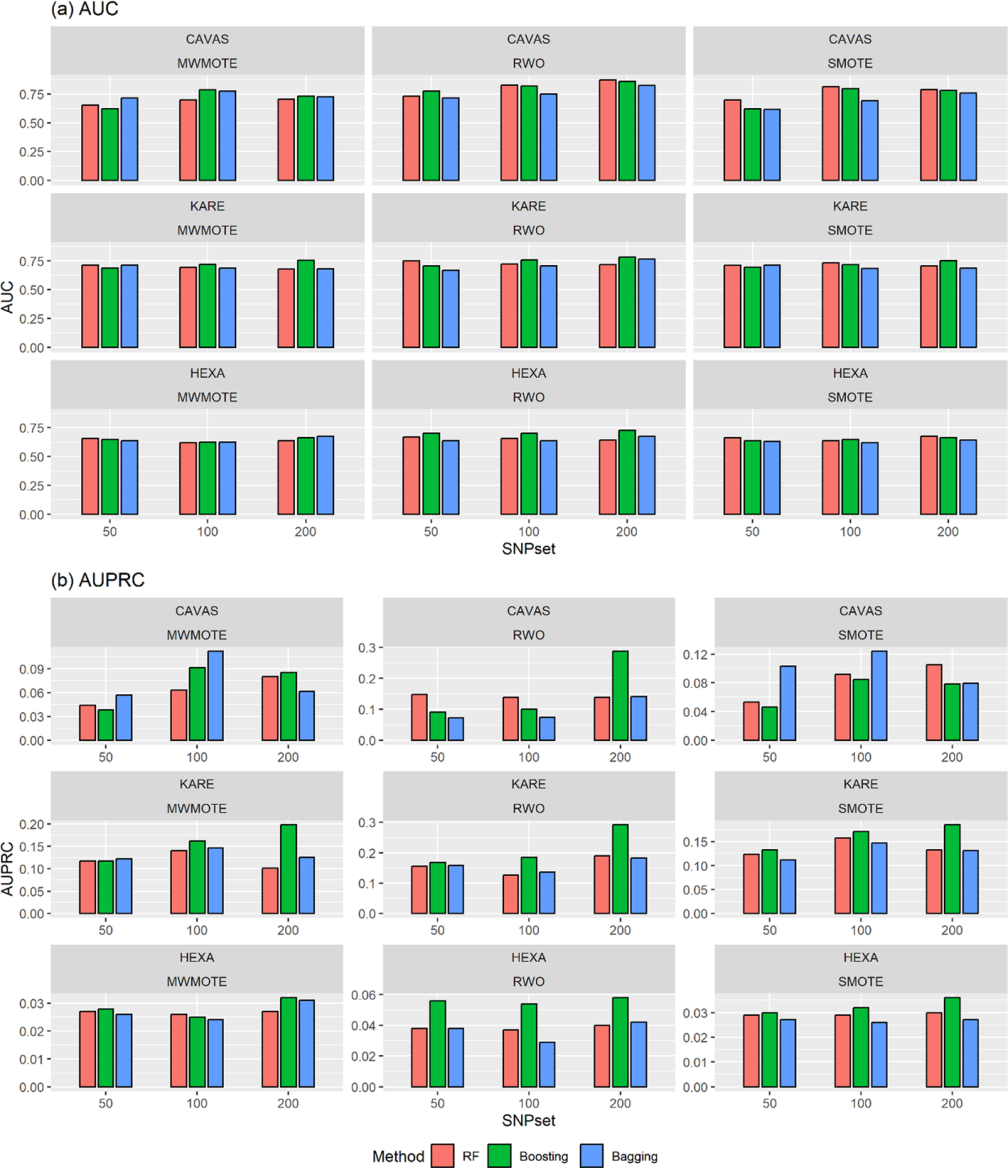
Comparison of **a** AUCs and **b** AUPRCs of RF, Boosting, and Bagging methods with oversampling algorithms on test datasets. CAVAS, Cardiovascular Disease Association Study; KARE, Korea Association Resource Study; HEXA, Health Examinees Study; AUC, area under the curve; AUPRC, area under the precision-recall curve; SNP, single-nucleotide polymorphism; RF, random forest; MWMOTE, majority weighted minority oversampling technique; RWO, random walk oversampling; SMOTE, synthetic minority oversampling technique

As shown in Table 4 and Additional file 1: Tables S3-S4, applying various oversampling algorithms results in improved AUCs for the machine learning methods compared to the results obtained from the analysis of the original data sets. However, it was confirmed that the performance of the penalized methods was inferior to that of using the original data set through oversampling algorithms. Especially, RF, boosting, and bagging methods outperformed penalized methods across the various top SNP sets in all cohorts. Furthermore, these methods showed that the prediction model′s performance improved as the number of SNP markers used increased. On the other hand, SVM, NB and KNN methods provided worse accuracy than the other methods for asthma under consideration. In Fig. 2a, the RWO algorithm showed the highest performance among oversampling algorithms for the RF, Boosting and Bagging methods. For instance, utilizing the top 200 SNPs in the CAVAS cohort, in the analysis using the RWO algorithm, the RF method established the best model with an AUC of 0.870, while in the analyses using the MWMOTE and SMOTE algorithms, the AUC values of the RF method were 0.704 and 0.787. respectively.

The improvement in AUPRC for the RF and boosting methods is more significant than the penalized and the other machine learning methods in Table 4 and Additional file 1: Tables S3-S4. For example, utilizing the top 200 SNPs in the KARE cohort, the boosting method established the best model using the RWO algorithm with an AUPRC of 0.293, while the AUPRC values of the Lasso and SCAD methods were 0.175 and 0.181. respectively. Figure 2b shows that the boosting method achieved higher AUPRCs than the RF and Bagging methods across various scenarios. Therefore, we can conclude that there is a difference in prediction model performance among oversampling algorithms, and the boosting method showed better prediction performance than penalized methods in the oversampling study.

### Functional annotations

Identified variants were annotated via ANNOVAR [63] from 1000 genomes using the human genome build 19 (hg19). We then conducted integrative functional annotation of the genetic variants via CADD [51] and DANN scores [52]. The CADD score is calculated into a scaled unit of 0 to 10 using the bottom 90% of all hg19 reference SNPs, while the top 10% to 1% occupy 10 to 20 units over. The DANN score ranges from 0 to 1, with a higher score more strongly suggesting deleterious variants. In order to reduce false positives, a threshold was adopted for each prediction algorithm (CADD ≥ 10 and DANN ≥ 0.6). Fifteen SNPs from 14 loci yielded consistent results in all algorithms (Additional file 1: Table S5). These variants were considered as prioritized putative SNPs, within the following corresponding genes: *RP3-348I23.3, PAK6, HOXB8, PPP3CA, GAPDHP56-RP11-401I19.1, LRBA, AC006145.1-CACNA2D1, COL4A3, RP11-138I17.1, RP11-1220K2.2, PDLIM2, LTA4H-RP11-256L6.4, KLF12,* and *SYNE2*. These genes were mainly related to asthma and lung disease [64–85].

### Comparison of computing time

**Fig. 3:**
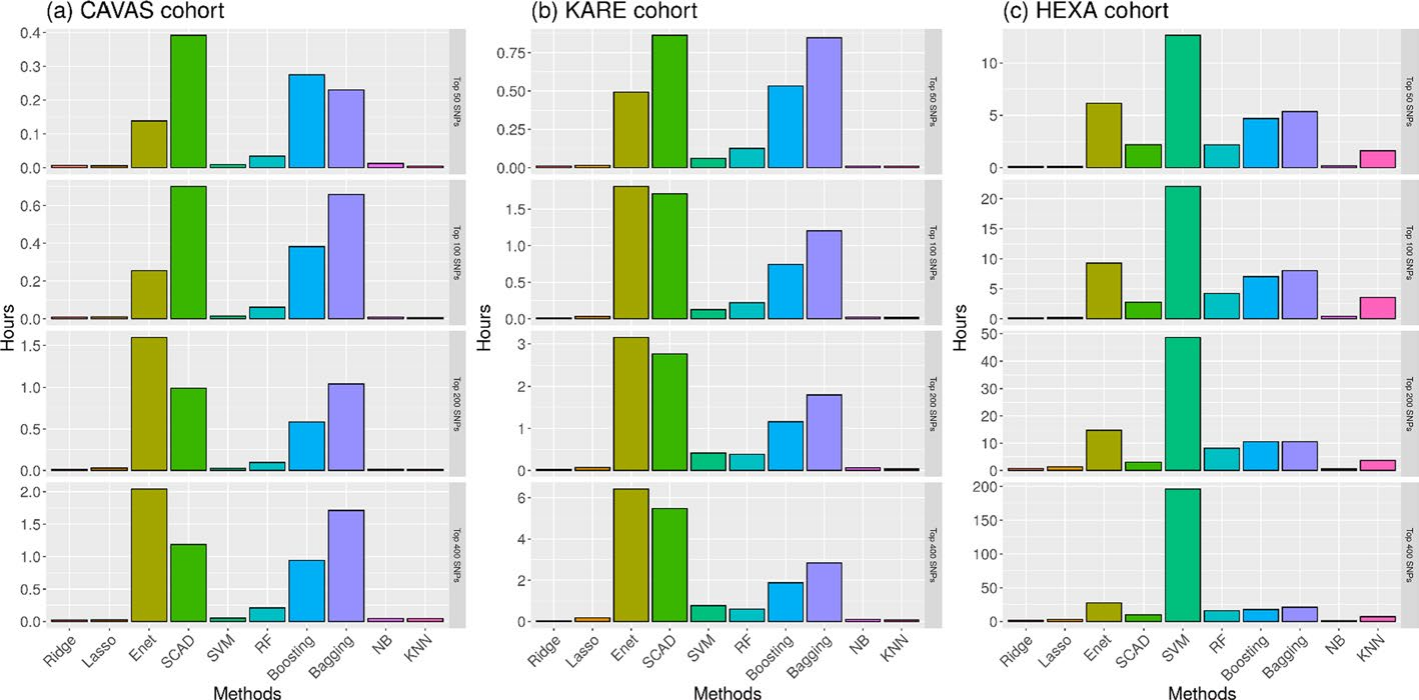
Bar plots showing the computing times for athma prediction methods in the **a** CAVAS cohort, **b** KARE cohort, and **c** HEXA cohort. CAVAS, Cardiovascular Disease Association Study; KARE, Korea Association Resource Study; HEXA, Health Examinees Study; SNP, single-nucleotide polymorphism; Lasso, least absolute shrinkage and selection operator; Enet, elastic net; SCAD, smoothly clipped absolute deviation; SVM, support vector machines; RF, random forest; NB, naïve Bayes; KNN, k-nearest neighbor classification

Computation was performed using an Intel Xeon Gold 6230 CPU @ 2.10 GHz, and the computation of the prediction process for each method was parallelized with five cores. Figure 3 shows that the computing time of the penalized and machine learning methods was calculated for five-fold CV. As shown in Fig. 3, prediction methods require greater computing time with an increasing number of SNPs. The ridge, Lasso, NB, and KNN methods have a faster computing speed than the other prediction methods. Interestingly, in the HEXA cohort, the SVM method was the slowest to predict the processing of large genetic and cohort datasets as computation time increased when obtaining parameters with various kernel options. Therefore, the SVM method heavily depends on sample size (*n*) rather than the SNPs (*p*). Comparing the computing time for the Lasso and Enet methods, which showed the best performance with regard to prediction accuracy, we determined that the Enet method takes 25 to 60 times more computing time than the Lasso method requires. Therefore, we can conclude that the Lasso method seems a more reasonable choice for reducing the computing time and maintaining the highest accuracy among prediction methods.

## Discussion

This study considered four penalized and six machine learning methods with three Korean trial cohorts that are computationally feasible for genome-wide SNP selection. Each method was used to construct a risk prediction model for asthma with a different number of SNPs. Then, five-fold CVs were used to fit the model, choose the tuning parameters, and evaluate the accuracy of predictive models. According to our results, penalized methods, such as Lasso and Enet, were generally more accurate than machine learning methods. Especially the Lasso method had the advantage of high computing speed in large genetic datasets compared to other existing methods. Furthermore, we applied three oversampling algorithms to deal with imbalance problems. In oversampling datasets, our results show that the boosting method generally performed superior to the penalized and the other machine learning methods.

Nevertheless, the current study has several limitations. First, the CV design tended to overestimate the prediction model’s accuracy. Therefore, we believe that the results should be interpreted with caution and may provide a general idea of the prediction accuracy of penalized and machine learning methods. Another limitation of this study is that various factors, such as filtering conditions for individuals or SNPs, covariates, and methods for choosing tuning parameters, can affect the accuracy of the final prediction model. In this study, we applied a one-standard deviation rule that could mitigate the overfitting problem for tuning the parameters [39]. However, this did not lead to a noticeable improvement in the results, indicating that the asthma-associated SNPs had small effect sizes and accounted for only a small fraction of the phenotypic variances. This explains why the penalized methods outperformed other machine-learning methods in our study. A third limitation of this study is that we did not consider most current prediction algorithms, such as deep learning and bootstrapping methods [86–89]. However, these approaches suffer from a heavy computational burden, which complicates their application on a genome-wide scale. As a final limitation of this study, an imbalanced dataset is recognized as a major cause of prediction performance degradation for machine learning methods. GWASs always suffer from the problem of an imbalanced dataset, having a sufficient control group and a limited case group. Such an imbalance issue can significantly challenge disease prediction [90–93]. To address the imbalance problem, we evaluated the performance of prediction methods by comprehensively considering AUC, precision, recall, F1-score, Cohen′s Kappa, BA, error rate, MCC, and AUPRC. Furthermore, we considered various oversampling techniques, such as MWMOTE, RWO, and SMOTE algorithms, to solve the imbalance problem. Our results show the differences between the various evaluation measures from overall scenarios. Although the recall value of the Lasso method was 0.947, the best score for the precision measure was only 0.548 using the ridge method when considering the top 400 SNP set in the CAVAS cohort. These results mean that our prediction model generates very few false negatives while generating many false positives. This consistently explains why the AUPRC measure can be a good performance evaluation in research on developing prediction models based on imbalanced data sets. However, we still have not completely solved the problem of many false positives generated in our prediction model. As one solution to these limitations, algorithm-level approaches can be considered. Algorithm-level approaches for addressing the imbalance problem have been adjusted to focus on learning the minority class by modifying the weight or cost of misclassification [94]. To explore the efficiency of algorithm-level approaches, we will study a large number of recently described methods in diverse genomic datasets.

## Conclusions

We compared penalized regression and machine learning methods (ridge, Lasso, Enet, SCAD, SVM, RF, boosting, bagging, NB, and KNN) for building asthma disease prediction models. Our results indicate that the former exhibited greater disease prediction accuracy. In particular, we recommend the Lasso method owing to its prediction accuracy and computing speed across all experiments. Nevertheless, in the case of imbalanced datasets, most prediction models do not perform properly, and various measures must be examined in combination as an indicator to evaluate model performance. Therefore, we applied various oversampling algorithms to examine the prediction model's performance comprehensively. Our results show that RWO algorithms performed better than the other oversampling algorithms and that RF and boosting methods provide better prediction performance than the existing methods for asthma disease under consideration. In function annotation studies, the top SNP sets were biologically associated with asthma or lung cancer based on functional prediction scores such as CADD and DANN. The predictive value of genetic variants as biomarkers should be further evaluated in related diseases or traits, and these results should be validated in other study populations.

## Materials and methods

### Study participants

This study was conducted using data from the KoGES consortium, including the CAVAS, KARE, and HEXA. The KoGES consortium is a large-scale longitudinal survey conducted by the Korea National Institute of Health from 2001–2010 to identify biomarkers and examine risk factors for common chronic diseases, such as obesity, diabetes, hypertension, and dyslipidemia, in South Korea. The detailed design and procedure of the KoGES consortium have been previously described [45].

In this study, there were a total of 72,296 participants, and participants with the following characteristics were excluded: did not provide asthma status (*n* = 5,182) (*n* = 5100 for the CAVAS cohort; *n* = 3 for the KARE cohort; and *n* = 79 for the HEXA cohort), did not provide allergy status (*n* = 5,187) (*n* = 5,100 for the CAVAS cohort; *n* = 3 for the KARE cohort; and *n* = 84 for the HEXA cohort), did not provide the smoking status (*n* = 265) (*n* = 4 for the CAVAS cohort; *n* = 67 for the KARE cohort; and *n* = 194 for the HEXA cohort), and did not provide BMI (*n* = 265) (*n* = 4 for the CAVAS cohort; *n* = 67 for the KARE cohort; and *n* = 194 for the HEXA cohort). A total of 66,857 participants (*n* = 3003 for the CAVAS cohort, *n* = 5,420 in the KARE cohort, and *n* = 58,434 for the HEXA cohort) were included in this analysis. The study was approved by the institutional review board of Hanyang University (IRB no. HYUIRB-202210–013).

### Genotyping and quality control

DNA samples from the three cohorts were genotyped using the Korea Biobank array (Korean Chip, KORV1.1), which was designed by the Center for Genome Science, Korea National Institute of Health, based on the platform of the UK Biobank Axiom array and manufactured by Affymetrix [47]. SNP imputation was performed with IMPUTE2 [95] using 1000 genomes from phase 3 data as a reference panel. Further details on genotype and quality control can be found in the work by Moon et al. [47]. The PLINK program (ver. 1.9) was used for quality control procedures [96]. Genetic variants with a high missing call rate > 0.05, missing rate per person > 0.05, low minor allele frequency < 0.05, and Hardy–Weinberg equilibrium *p*-values ≤ 1 × 10^–5^ were excluded. After quality control, 5,166,416 autosomal SNPs remained for association analysis.

### SNP prescreening

In GWASs, a logistic regression model is one of the most commonly used models to test for associations between genotype and phenotype while adjusting for a set of covariates. Therefore, we conducted a single SNP logistic regression analysis to select an effective list of SNPs for testing the model as follows:

1 $$\text{logit}\left( {\pi (\mathbf{X}, \mathbf{COV})} \right) = \log \frac{{P\left( {\mathbf{Y} = 1 | \mathbf{X}, \mathbf{COV}} \right)}}{{1 - P\left( {\mathbf{Y} = 1 | \mathbf{X}, \mathbf{COV}} \right)}} = \mathbf{X}\boldsymbol{\beta} + \mathbf{COV} \boldsymbol{\gamma} ,$$

where **Y** is an *n*-dimensional vector of zeroes and ones (control = 0, case = 1), and **X** is a vector of genotypes for individuals. The genetic SNP values were encoded in three different numbers (AA = 0, Aa = 1, aa = 2), where “A” and “a” indicate major and minor alleles, respectively. **COV** is an *n* × 16 matrix of covariates, representing sex, age, BMI, smoking status, allergy status, and the top 10 principal components (PCs) (including a column of ones for the intercept). We calculated the 10 PCs using train sets for autosomal chromosomes. Since the number of SNPs seems related to predictive performance, we selected SNP sets for each cohort based on the order of *p*-values (50, 100, 200, and 400 SNPs).

### Stratified *k*-fold cross-validation

**Fig. 4:**
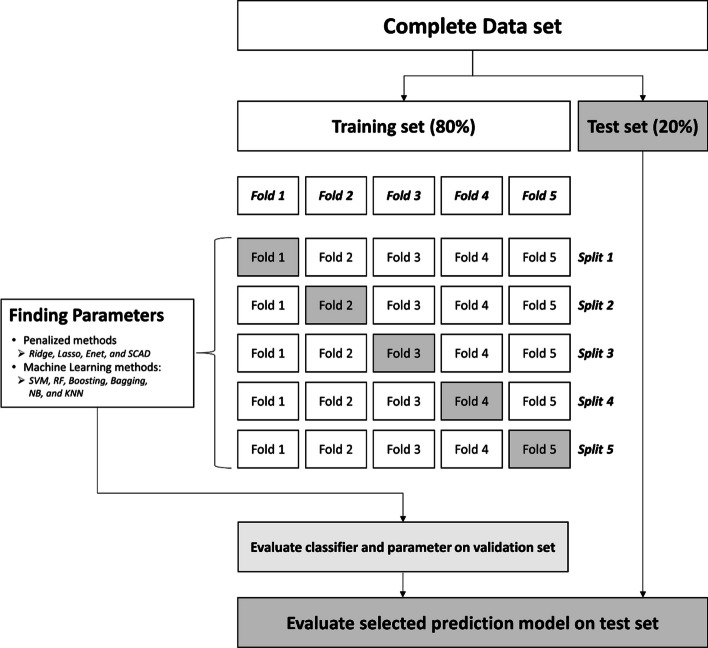
Flowchart of prediction model construction and evaluation. SNP, single-nucleotide polymorphism; Lasso, least absolute shrinkage and selection operator; Enet, elastic net; SCAD, smoothly clipped absolute deviation; SVM, support vector machines; RF, random forest; NB, naïve Bayes; KNN, k-nearest neighbor classification; CV, cross-validation

As per GWAS data, the case group was much smaller than the control group (Table 1). If this condition is not considered, prediction methods may be biased and trained only based on the control group. Therefore, we applied a stratified *k*-fold CV method that enables each fold to have the same proportion of cases and controls. A *k* value of 5 was used to evaluate the accuracy of the disease prediction methods. Figure 4 shows a flowchart of prediction model construction and evaluation. The GWAS data were first randomly divided into training (80%) and test (20%) sets, taking into account the ratio of cases and controls. Next, a stratified *k*-fold CV was performed on the training set and repeated five times after data shuffling.

### Penalized methods

A number of penalized regression methods have been suggested recently, and we considered four of these in the current study: ridge [33], Lasso [36], Enet [37], and SCAD [38]. The penalized logistic regression coefficients were determined by minimizing the log-likelihood function *l*(***β****,****γ***) over *n* observations.

2 $$l(\boldsymbol{\beta}, \boldsymbol{\gamma} ) = - \mathop \sum \limits_{i = 1}^{n} \left\{ {y_{i} \log \left( {\pi \left( {\mathbf{X}_{i} , \mathbf{COV}_{i} } \right)} \right) + \left( {1 - y_{i} } \right)\log \left( {1 - \pi \left( {\mathbf{X}_{i} , \mathbf{COV}_{i} } \right)} \right)} \right\} + P_{\lambda } \left( {\boldsymbol{\beta} ,\boldsymbol{\gamma} } \right),$$

where *P*_*λ*_(***β***, ***γ***) is a general penalty function with the tuning parameter *λ*. We used fivefold CV to find the value for the tuning parameter, *λ*, selected via the one standard deviation rule [39]. That is, selecting the model that produces a mean squared error (MSE) within one standard deviation of the minimal MSE. Many researchers used penalized methods to investigate variable selection and classification problems considering linkage disequilibrium among predictor SNPs in GWASs [40–44]. We used the *glmnet* R-package to implement the penalized methods [97].

The ridge regression method proposed by Hoerl and Kennard [33] employs a penalty term that regularizes the regression coefficient through an *L*_2_–norm penalized least-square criterion [i.e., *P*_*λ*_(***β***, ***γ***) = *λ*(*∑ β*^2^ + *∑ γ*^2^)]. In ridge regression, the tuning parameter controls the amount of shrinkage. If it is set to a higher value, the ridge regression shrinks the estimated coefficients toward zero. However, the estimated coefficients will not be set to zero, and the intercept term is not penalized.

The Lasso method proposed by Tibshirani [36] employs the *L*_1_–norm penalized least-square criterion [i.e., *P*_*λ*_(***β***, ***γ***) = *λ*(*∑* |*β*|+ *∑* |*γ*|)]. Unlike the ridge method, the Lasso method performs variable selection, with higher values of *λ* leading some coefficients of the model toward zero. Hence, the Lasso method has the advantage of reducing overestimation by simultaneously performing estimation and variable selection of risk predictors.

The Enet method proposed by Zou and Hastie [37] is a hybrid of ridge and Lasso penalties, defined as *P*_*λ*_(***β***, ***γ***) = *λ*[(1 – *α*)(*∑ β*^2^ + *∑ γ*^2^) + *α*(*∑* |*β*|+ *∑* |*γ*|)], where *α* is the penalty weight of a value between 0 and 1. If *α* is set to zero, the Enet method is equivalent to the ridge method. Otherwise, setting *α* close to 1 makes the Enet method identical to the Lasso method. Empirical simulation studies and real data analysis have suggested that the Enet method often outperforms Lasso in data with highly correlated risk predictors [37].

The SCAD penalty proposed by Fan and Li [38] is defined as follows:

3 $$\frac{{\partial P\left( {\boldsymbol{\beta} , \gamma } \right)}}{{\partial \left( {\boldsymbol{\beta} , \gamma } \right)}} = \sum \lambda \left\{ {I\left( {\left| {\beta , \gamma } \right| \le \lambda } \right) + \frac{{\left( {a\lambda - \left| {\beta , \gamma } \right|} \right)_{ + } }}{{\left( {a - 1} \right)}}I\left( {\left| {\beta , \gamma } \right| > \lambda } \right)} \right\},$$

where *a* is a fixed constant larger than 2, the notation (‧)_+_ stands for the positive part, and *I*(‧) denotes the indicator function. The SCAD method produces the same behavior as the Lasso penalty for small coefficients but assigns a constant penalty for large coefficients. Hence, the SCAD method can reduce the estimation bias and achieve a stable model of optimal subset selection.

### Machine learning methods

Various machine learning methods have been proposed, and we consider six penalized methods in this study: SVM [23], RF [25], boosting [26], bagging [27], NB [28, 98], and KNN [29]. We used fivefold CV to find out the optimal kernel and parameters of machine learning methods.

The SVM method, introduced by Vapnik [23], is widely used as a supervised learning algorithm to solve classification problems, with successful application in various bioinformatics tasks. The SVM method is based on finding the optimal hyperplane that best separates data points into two classes. However, this method does not provide a biological interpretation of each predictor variable in an SNP set. We implemented the SVM method with sigmoid, linear, polynomial, and radial kernel functions using the R-package *e1071* [99].

The RF method, proposed by Breiman [25], is an ensemble classification approach that generates bootstrap sampling using sets of random decision trees for decision making and voting in classification problems. The RF method provides the relative importance of each feature in a prediction model. This method has been successfully applied in genetics research [100–102]. We used the R-package *randomForest* with default settings [103].

The boosting method, proposed by Schapire [26], is one of the most popular approaches for reducing variance and bias in ensemble machine learning. The basic principle of the boosting method is to iteratively assemble multiple weak learning models in order to establish a robust model that is markedly better in prediction than any of the single models. Many researchers have demonstrated the performance of the boosting method and its optimization for genomic selection, gene interaction, and genetic disease diagnosis [104–107]. We used the R-package *ada,* including discrete, real, and gentle type functions [108].

The bagging method, proposed by Breiman [27], is an ensemble algorithm used to generate many predictors and obtain an aggregated predictor to be used for statistical classification. The bagging method effectively reduces the variance of a model, increases accurate estimates, and prevents overfitting. Many researchers employed the bagging method, demonstrating its performance in bioinformatics classification and gene selection [109–111]. We used the R-package *ipred* with different nbagg value options (nbagg = 25, 50, 100, and 200) [112].

The NB method based on Bayes′ theorem [28, 98] is a supervised learning algorithm for solving classification problems. The NB method is a probabilistic classifier using the assumption of conditional independence between the different variables in a given dataset. It was previously employed to improve the performance of gene selection and classification based on gene expression [113–116]. We used the R-package *e1071* with default settings [99].

The KNN method proposed by Cover and Hart [29] is one of the most common pattern recognition algorithms. The main idea of the KNN method is to extract *k* closest data with input data existing in close. The KNN method is also helpful in gene selection, cancer classification, and diagnosis based on gene expression [117–120]. We used the R-package *caret* with default settings [57].

### Evaluation of disease risk prediction models

We compared and evaluated the performance of predictive models on imbalanced datasets based on precision, recall, F1-score, Cohen′s Kappa, BA, error rate, MCC, AUC, and AUPRC. The performance metrics can be calculated from the number of true positives (TPs), false positives (FPs), false negatives (FNs), and true negatives (TNs). Precision, also known as the positive predictive value, is calculated via the following formula:

4 $${\text{Precision }} = \frac{{{\text{TP}}}}{{{\rm{TP}} + {\rm{FP}}}}.$$

The recall or sensitivity can be calculated via the following formula:

5 $${\text{Recall}} = \frac{{{\text{TP}}}}{{{\rm{TP}} + {\rm{FN}}}}.$$

The F1-score is a combined measure of precision and recall, which can be determined via the following formula:

6 $${\text{F1-score}} = \frac{{2 \times {\text{Precision}} \times {\text{Recall}}}}{{{\rm{Precision}} + {\rm{Recall}}}}.$$

Cohen′s Kappa is commonly used to quantify the degree of agreement between raters on a nominal scale and can be calculated via the following formula:

7 $${\text{Kappa }} = \frac{{\frac{{{\text{TP}} + {\text{TN}}}}{{{\rm{TP}} + {\rm{TN}} + {\rm{FP}} + {\rm{FN}}}}}}{{\frac{{{\text{TP}} + {\rm{TN}}}}{{{\rm{TP}} + {\rm{TN}} + {\rm{FP}} + {\rm{FN}}}} + \frac{{\left( {{\text{TP}} + {\rm{TN}}} \right) \times \left( {{\rm{FP}} + {\text{FN}}} \right)}}{{2 \times \left( {{\rm{TP }} \times {\text{TN}} - {\rm{FP}} \times {\rm{FN}}} \right)}}}}.$$

The BA is the average of sensitivity and specificity that is defined via the following formula:

8 $${\text{BA}} = \frac{{\frac{{{\text{TP}}}}{{{\rm{TP}} + {\rm{FN}}}} + \frac{{{\text{TN}}}}{{{\rm{TN}} + {\rm{FP}}}}}}{2}.$$

The error rate represents the ratio of incorrect predictions among a total number of results and can be calculated via the following formula:

9 $${\text{Error rate }} = \frac{{{\text{FP}} + {\rm{ FN}}}}{{{\rm{TP}} + {\text{TN}} + {\rm{FP}} + {\rm{FN}}}}.$$

The MCC calculates the Pearson correlation coefficient between observed and predicted classifications that range from -1 (worst value) to 1 (best value). The MCC is defined via the following formula:

10 $${\text{MCC }} = \frac{{{\text{TP}} \times {\rm{TN}} - {\rm{FP}} \times {\text{FN}}}}{{\sqrt {\left( {{\rm{TP}} + {\rm{ FP}}} \right)\left( {{\text{TP}} + {\text{FN}}} \right)\left( {{\rm{TN}} + {\rm{FP}}} \right)\left( {\text{TN + FN}} \right)} }}.$$

The AUC of the ROC is widely used as an overall summary measure of discriminative accuracy in binary classification [121, 122]. ROC curve indicates the relationship between the true positive and false positive rates for all possible threshold values. For example, an AUC score close to 0.5 corresponds to random chance, whereas a maximum value of 1.0 implies perfect discriminatory power.

The AUPRC is an informative evaluation measure, especially on imbalanced biological and medical datasets [123–125]. The precision-recall curve (PRC) is composed of the recall (*x*-axis) and the precision (*y*-axis) for different probability thresholds [126]. Unlike the baseline of the ROC curve, which is fixed at 0.5, the baseline of PRC is determined by the ratio of positives (P) and negatives (N) as y = P/(P + N). For instance, the baseline of PRC is y = 0.5 in the case of balanced data. However, it is changed to y = 0.09 in the imbalanced data with a P:N ratio of 1:10. Thus, the AUC score is constant regardless of the positive rate, but the AUPRC decreases accordingly as the positive rate decreases. For example, when the positive rate is 0.01, an AUPRC of 0.10 means that the prediction model's performance is ten times better than the baseline of 0.01.

### Supplementary Information

**Additional file 1.** Supplementary tables.

## Data Availability

The CAVAS, KARE, and HEXA Korean Chip (KORV1.1) datasets are a part of KoGES consortium, and are available upon approval of the genome center in Korea National Institute of Health (https://biobank.nih.go.kr/). Any inquiries should be sent to biobank@korea.kr.

## Abbreviations

GWASs: Genome-wide association studies
SNPs: Single nucleotide polymorphisms
SVM: Support vector machine
RF: Random forest
NB: Naïve Bayes
KNN: K-nearest neighbor classification
Lasso: Least absolute shrinkage and selection operator
Enet: Elastic net
SCAD: Smoothly clipped absolute deviation
KoGES: Korean Genome and Epidemiology Study
AUC: Area under the curve
ROC: Receiver operating characteristic
BA: Balanced accuracy
MCC: Matthews correlation coefficient
AUPRC: Area under the precision-recall curve
MWMOTE: Majority weighted minority oversampling technique
RWO: Random walk oversampling
SMOTE: Synthetic minority oversampling technique
CADD: Combined Annotation-Dependent Depletion
DANN: Deleterious annotation of genetic variants using neural networks
CAVAS: Cardiovascular Disease Association Study
KARE: Korea Association Resource Study
HEXA: Health Examinees Study
BMI: Body mass index
CI: Confidence interval
CV: Cross-validation
PCs: Principal components
MSE: Mean squared error
TP: True positive
FP: False positive
FN: False negative
TN: True negative
PRC: Precision-recall curve
P: Positives
N: Negatives
