# Supplementary material for: Evaluation of penalized and machine learning methods for asthma disease prediction in the Korean Genome and Epidemiology Study (KoGES)
Source: BMC Bioinformatics. 2024 Feb 2;25:56. doi: 10.1186/s12859-024-05677-x (PMC10837879; doi:10.1186/s12859-024-05677-x)
Supplement: Supplementary file 1 — Additional file 1. Supplementary tables. [file 12859_2024_5677_MOESM1_ESM.docx]

Table S1. Performance evaluation metrics for prediction methods in the KARE cohort using the test dataset

| **# of SNPs** | **Metrics** | **Ridge** | **Lasso** | **Enet** | **SCAD** | **SVM** | **RF** | **Boosting** | **Bagging** | **NB** | **KNN** |
| --- | --- | --- | --- | --- | --- | --- | --- | --- | --- | --- | --- |
| **50** | **AUC** | 0.755 | 0.763 | 0.761 | 0.763 | 0.628 | 0.643 | 0.648 | 0.577 | 0.658 | 0.491 |
|  | **Precision** | 0.115 | 0.108 | 0.128 | 0.129 | 0.092 | 0.089 | 0.098 | 0.065 | 0.087 | 0.051 |
|  | **Recall** | 0.778 | 0.841 | 0.714 | 0.698 | 0.603 | 0.619 | 0.571 | 0.683 | 0.889 | 0.159 |
|  | **F1-score** | 0.201 | 0.191 | 0.217 | 0.217 | 0.160 | 0.155 | 0.168 | 0.119 | 0.158 | 0.078 |
|  | **Cohen**′**s Kappa** | 0.111 | 0.098 | 0.131 | 0.132 | 0.065 | 0.059 | 0.076 | 0.015 | 0.058 | -0.011 |
|  | **Balanced accuracy** | 0.705 | 0.706 | 0.707 | 0.703 | 0.618 | 0.613 | 0.624 | 0.540 | 0.655 | 0.489 |
|  | **Error rate** | 0.360 | 0.414 | 0.300 | 0.293 | 0.369 | 0.392 | 0.330 | 0.586 | 0.552 | 0.220 |
|  | **MCC** | 0.196 | 0.194 | 0.207 | 0.205 | 0.114 | 0.108 | 0.123 | 0.038 | 0.148 | -0.014 |
|  | **AUPRC** | 0.146 | 0.153 | 0.151 | 0.141 | 0.102 | 0.090 | 0.098 | 0.110 | 0.102 | 0.075 |
| **100** | **AUC** | 0.793 | 0.803 | 0.802 | 0.799 | 0.636 | 0.643 | 0.726 | 0.611 | 0.667 | 0.553 |
|  | **Precision** | 0.126 | 0.128 | 0.127 | 0.124 | 0.097 | 0.089 | 0.105 | 0.066 | 0.092 | 0.058 |
|  | **Recall** | 0.825 | 0.825 | 0.825 | 0.841 | 0.651 | 0.762 | 0.825 | 0.746 | 0.810 | 1.000 |
|  | **F1-score** | 0.218 | 0.222 | 0.220 | 0.216 | 0.169 | 0.159 | 0.187 | 0.121 | 0.165 | 0.110 |
|  | **Cohen**′**s Kappa** | 0.130 | 0.135 | 0.133 | 0.127 | 0.075 | 0.062 | 0.093 | 0.016 | 0.068 | 0.000 |
|  | **Balanced accuracy** | 0.735 | 0.739 | 0.738 | 0.737 | 0.638 | 0.640 | 0.696 | 0.547 | 0.658 | 0.500 |
|  | **Error rate** | 0.344 | 0.337 | 0.340 | 0.355 | 0.373 | 0.467 | 0.418 | 0.630 | 0.476 | 0.942 |
|  | **MCC** | 0.227 | 0.231 | 0.230 | 0.227 | 0.133 | 0.131 | 0.184 | 0.046 | 0.148 | 0.000 |
|  | **AUPRC** | 0.169 | 0.171 | 0.172 | 0.169 | 0.086 | 0.093 | 0.150 | 0.095 | 0.095 | 0.083 |
| **200** | **AUC** | 0.858 | 0.867 | 0.863 | 0.869 | 0.866 | 0.716 | 0.720 | 0.710 | 0.747 | 0.554 |
|  | **Precision** | 0.184 | 0.148 | 0.178 | 0.147 | 0.180 | 0.105 | 0.103 | 0.143 | 0.100 | 0.058 |
|  | **Recall** | 0.825 | 0.937 | 0.841 | 0.952 | 0.905 | 0.778 | 0.778 | 0.651 | 0.873 | 1.000 |
|  | **F1-score** | 0.301 | 0.255 | 0.294 | 0.255 | 0.301 | 0.186 | 0.182 | 0.235 | 0.179 | 0.110 |
|  | **Cohen**′**s Kappa** | 0.227 | 0.172 | 0.219 | 0.171 | 0.226 | 0.093 | 0.089 | 0.154 | 0.083 | 0.000 |
|  | **Balanced accuracy** | 0.799 | 0.802 | 0.801 | 0.806 | 0.825 | 0.685 | 0.681 | 0.705 | 0.693 | 0.500 |
|  | **Error rate** | 0.223 | 0.318 | 0.235 | 0.324 | 0.245 | 0.397 | 0.405 | 0.247 | 0.466 | 0.942 |
|  | **MCC** | 0.319 | 0.293 | 0.315 | 0.295 | 0.335 | 0.175 | 0.170 | 0.218 | 0.181 | 0.000 |
|  | **AUPRC** | 0.296 | 0.303 | 0.310 | 0.295 | 0.254 | 0.149 | 0.147 | 0.139 | 0.132 | 0.072 |
| **400** | **AUC** | 0.897 | 0.903 | 0.903 | 0.900 | 0.872 | 0.756 | 0.767 | 0.685 | 0.784 | 0.574 |
|  | **Precision** | 0.204 | 0.229 | 0.229 | 0.216 | 0.161 | 0.120 | 0.156 | 0.074 | 0.115 | 0.120 |
|  | **Recall** | 0.841 | 0.841 | 0.841 | 0.825 | 0.905 | 0.683 | 0.730 | 0.889 | 0.873 | 0.270 |
|  | **F1-score** | 0.328 | 0.361 | 0.361 | 0.342 | 0.274 | 0.204 | 0.258 | 0.137 | 0.204 | 0.166 |
|  | **Cohen**′**s Kappa** | 0.259 | 0.296 | 0.296 | 0.275 | 0.195 | 0.116 | 0.179 | 0.034 | 0.112 | 0.093 |
|  | **Balanced accuracy** | 0.819 | 0.833 | 0.833 | 0.820 | 0.807 | 0.686 | 0.744 | 0.603 | 0.730 | 0.574 |
|  | **Error rate** | 0.200 | 0.174 | 0.174 | 0.185 | 0.279 | 0.310 | 0.245 | 0.649 | 0.397 | 0.158 |
|  | **MCC** | 0.350 | 0.381 | 0.381 | 0.360 | 0.307 | 0.185 | 0.256 | 0.105 | 0.217 | 0.102 |
|  | **AUPRC** | 0.403 | 0.408 | 0.408 | 0.421 | 0.327 | 0.171 | 0.200 | 0.107 | 0.152 | 0.084 |

Table S2. Performance evaluation metrics for prediction methods in the HEXA cohort using the test dataset

| **# of SNPs** | **Metrics** | **Ridge** | **Lasso** | **Enet** | **SCAD** | **SVM** | **RF** | **Boosting** | **Bagging** | **NB** | **KNN** |
| --- | --- | --- | --- | --- | --- | --- | --- | --- | --- | --- | --- |
| **50** | **AUC** | 0.706 | 0.714 | 0.716 | 0.716 | 0.546 | 0.600 | 0.636 | 0.523 | 0.672 | 0.520 |
|  | **Precision** | 0.036 | 0.038 | 0.039 | 0.035 | 0.020 | 0.032 | 0.028 | 0.018 | 0.034 | 0.026 |
|  | **Recall** | 0.672 | 0.631 | 0.615 | 0.682 | 0.533 | 0.400 | 0.513 | 0.713 | 0.544 | 0.113 |
|  | **F1-score** | 0.068 | 0.072 | 0.073 | 0.066 | 0.039 | 0.060 | 0.053 | 0.035 | 0.064 | 0.042 |
|  | **Cohen**′**s Kappa** | 0.037 | 0.042 | 0.042 | 0.035 | 0.007 | 0.030 | 0.022 | 0.002 | 0.033 | 0.015 |
|  | **Balanced accuracy** | 0.682 | 0.681 | 0.677 | 0.680 | 0.547 | 0.598 | 0.606 | 0.521 | 0.640 | 0.520 |
|  | **Error rate** | 0.308 | 0.271 | 0.263 | 0.321 | 0.440 | 0.210 | 0.305 | 0.665 | 0.267 | 0.086 |
|  | **MCC** | 0.101 | 0.104 | 0.103 | 0.098 | 0.024 | 0.062 | 0.059 | 0.011 | 0.081 | 0.020 |
|  | **AUPRC** | 0.058 | 0.058 | 0.059 | 0.057 | 0.025 | 0.027 | 0.036 | 0.018 | 0.050 | 0.019 |
| **100** | **AUC** | 0.718 | 0.733 | 0.733 | 0.726 | 0.502 | 0.621 | 0.657 | 0.590 | 0.670 | 0.522 |
|  | **Precision** | 0.042 | 0.042 | 0.031 | 0.035 | 0.017 | 0.027 | 0.034 | 0.023 | 0.036 | 0.027 |
|  | **Recall** | 0.549 | 0.605 | 0.759 | 0.703 | 0.662 | 0.518 | 0.554 | 0.559 | 0.508 | 0.118 |
|  | **F1-score** | 0.078 | 0.078 | 0.060 | 0.066 | 0.034 | 0.051 | 0.063 | 0.045 | 0.067 | 0.043 |
|  | **Cohen**′**s Kappa** | 0.048 | 0.048 | 0.029 | 0.036 | 0.001 | 0.020 | 0.033 | 0.013 | 0.036 | 0.017 |
|  | **Balanced accuracy** | 0.668 | 0.685 | 0.681 | 0.686 | 0.514 | 0.599 | 0.642 | 0.580 | 0.637 | 0.522 |
|  | **Error rate** | 0.217 | 0.238 | 0.394 | 0.330 | 0.628 | 0.323 | 0.273 | 0.400 | 0.238 | 0.087 |
|  | **MCC** | 0.104 | 0.110 | 0.095 | 0.101 | 0.008 | 0.054 | 0.082 | 0.042 | 0.083 | 0.022 |
|  | **AUPRC** | 0.053 | 0.060 | 0.060 | 0.054 | 0.018 | 0.041 | 0.040 | 0.027 | 0.040 | 0.019 |
| **200** | **AUC** | 0.747 | 0.762 | 0.762 | 0.761 | 0.543 | 0.586 | 0.664 | 0.540 | 0.692 | 0.522 |
|  | **Precision** | 0.038 | 0.045 | 0.048 | 0.037 | 0.019 | 0.020 | 0.036 | 0.023 | 0.034 | 0.027 |
|  | **Recall** | 0.667 | 0.621 | 0.605 | 0.718 | 0.836 | 0.733 | 0.446 | 0.256 | 0.600 | 0.113 |
|  | **F1-score** | 0.072 | 0.084 | 0.089 | 0.071 | 0.037 | 0.039 | 0.067 | 0.043 | 0.064 | 0.044 |
|  | **Cohen**′**s Kappa** | 0.042 | 0.055 | 0.060 | 0.040 | 0.004 | 0.007 | 0.038 | 0.012 | 0.033 | 0.017 |
|  | **Balanced accuracy** | 0.691 | 0.699 | 0.701 | 0.701 | 0.549 | 0.566 | 0.623 | 0.537 | 0.654 | 0.522 |
|  | **Error rate** | 0.286 | 0.225 | 0.207 | 0.315 | 0.728 | 0.597 | 0.206 | 0.193 | 0.294 | 0.082 |
|  | **MCC** | 0.107 | 0.121 | 0.126 | 0.110 | 0.029 | 0.034 | 0.078 | 0.024 | 0.086 | 0.022 |
|  | **AUPRC** | 0.074 | 0.083 | 0.082 | 0.080 | 0.019 | 0.024 | 0.047 | 0.025 | 0.037 | 0.019 |
| **400** | **AUC** | 0.831 | 0.836 | 0.836 | 0.833 | 0.685 | 0.609 | 0.720 | 0.579 | 0.728 | 0.518 |
|  | **Precision** | 0.053 | 0.048 | 0.048 | 0.053 | 0.033 | 0.021 | 0.036 | 0.033 | 0.029 | 0.017 |
|  | **Recall** | 0.738 | 0.785 | 0.785 | 0.744 | 0.595 | 0.805 | 0.662 | 0.251 | 0.795 | 1.000 |
|  | **F1-score** | 0.099 | 0.091 | 0.091 | 0.099 | 0.063 | 0.042 | 0.068 | 0.058 | 0.055 | 0.033 |
|  | **Cohen**′**s Kappa** | 0.070 | 0.061 | 0.061 | 0.070 | 0.032 | 0.009 | 0.038 | 0.029 | 0.024 | 0.000 |
|  | **Balanced accuracy** | 0.758 | 0.761 | 0.761 | 0.759 | 0.649 | 0.589 | 0.680 | 0.563 | 0.668 | 0.500 |
|  | **Error rate** | 0.224 | 0.262 | 0.262 | 0.227 | 0.298 | 0.619 | 0.303 | 0.136 | 0.456 | 0.983 |
|  | **MCC** | 0.156 | 0.150 | 0.150 | 0.156 | 0.083 | 0.047 | 0.100 | 0.048 | 0.086 | 0.000 |
|  | **AUPRC** | 0.097 | 0.105 | 0.105 | 0.107 | 0.039 | 0.025 | 0.048 | 0.027 | 0.043 | 0.020 |

Table S3. Comparison of the prediction performance of methods with oversampling algorithms in the KARE cohort using the test dataset

| **Algorithm** | **# of**  **SNP** | **Metrics** | **Ridge** | **Lasso** | **Enet** | **SCAD** | **SVM** | **RF** | **Boosting** | **Bagging** | **NB** | **KNN** |
| --- | --- | --- | --- | --- | --- | --- | --- | --- | --- | --- | --- | --- |
| **MWMOTE** | **50** | **AUC** | 0.594 | 0.634 | 0.634 | 0.630 | 0.519 | 0.712 | 0.687 | 0.714 | 0.582 | 0.557 |
|  |  | **Precision** | 0.090 | 0.098 | 0.103 | 0.095 | 0.036 | 0.131 | 0.112 | 0.118 | 0.078 | 0.119 |
|  |  | **Recall** | 0.490 | 0.608 | 0.608 | 0.608 | 0.137 | 0.615 | 0.750 | 0.731 | 0.569 | 0.196 |
|  |  | **F1-score** | 0.152 | 0.169 | 0.177 | 0.164 | 0.057 | 0.215 | 0.195 | 0.204 | 0.138 | 0.148 |
|  |  | **Cohen′s Kappa** | 0.059 | 0.076 | 0.085 | 0.070 | -0.040 | 0.130 | 0.102 | 0.112 | 0.038 | 0.081 |
|  |  | **Balanced accuracy** | 0.591 | 0.630 | 0.639 | 0.623 | 0.454 | 0.677 | 0.687 | 0.692 | 0.575 | 0.553 |
|  |  | **Error rate** | 0.321 | 0.351 | 0.333 | 0.363 | 0.266 | 0.268 | 0.369 | 0.342 | 0.419 | 0.133 |
|  |  | **MCC** | 0.092 | 0.127 | 0.138 | 0.120 | -0.052 | 0.187 | 0.181 | 0.189 | 0.072 | 0.084 |
|  |  | **AUPRC** | 0.075 | 0.097 | 0.093 | 0.094 | 0.059 | 0.117 | 0.117 | 0.122 | 0.119 | 0.079 |
|  | **100** | **AUC** | 0.596 | 0.646 | 0.658 | 0.652 | 0.531 | 0.693 | 0.718 | 0.686 | 0.514 | 0.554 |
|  |  | **Precision** | 0.082 | 0.103 | 0.104 | 0.100 | 0.051 | 0.137 | 0.118 | 0.109 | 0.065 | 0.073 |
|  |  | **Recall** | 0.569 | 0.549 | 0.569 | 0.627 | 0.314 | 0.569 | 0.686 | 0.647 | 0.588 | 0.588 |
|  |  | **F1-score** | 0.143 | 0.173 | 0.176 | 0.173 | 0.088 | 0.221 | 0.201 | 0.187 | 0.118 | 0.129 |
|  |  | **Cohen′s Kappa** | 0.045 | 0.082 | 0.085 | 0.079 | -0.014 | 0.139 | 0.112 | 0.096 | 0.013 | 0.027 |
|  |  | **Balanced accuracy** | 0.585 | 0.624 | 0.632 | 0.637 | 0.476 | 0.672 | 0.683 | 0.659 | 0.531 | 0.559 |
|  |  | **Error rate** | 0.400 | 0.309 | 0.313 | 0.354 | 0.381 | 0.236 | 0.321 | 0.331 | 0.519 | 0.466 |
|  |  | **MCC** | 0.082 | 0.126 | 0.133 | 0.134 | -0.023 | 0.189 | 0.181 | 0.157 | 0.029 | 0.056 |
|  |  | **AUPRC** | 0.087 | 0.125 | 0.126 | 0.110 | 0.060 | 0.140 | 0.162 | 0.146 | 0.065 | 0.072 |
|  | **200** | **AUC** | 0.576 | 0.734 | 0.730 | 0.745 | 0.634 | 0.677 | 0.754 | 0.680 | 0.436 | 0.533 |
|  |  | **Precision** | 0.075 | 0.113 | 0.112 | 0.109 | 0.085 | 0.103 | 0.129 | 0.111 | 0.027 | 0.067 |
|  |  | **Recall** | 0.627 | 0.765 | 0.765 | 0.843 | 0.843 | 0.706 | 0.745 | 0.706 | 0.078 | 0.863 |
|  |  | **F1-score** | 0.134 | 0.197 | 0.195 | 0.192 | 0.154 | 0.180 | 0.220 | 0.191 | 0.040 | 0.124 |
|  |  | **Cohen′s Kappa** | 0.033 | 0.106 | 0.103 | 0.098 | 0.053 | 0.087 | 0.133 | 0.100 | -0.053 | 0.017 |
|  |  | **Balanced accuracy** | 0.573 | 0.695 | 0.692 | 0.705 | 0.638 | 0.662 | 0.715 | 0.676 | 0.450 | 0.556 |
|  |  | **Error rate** | 0.475 | 0.366 | 0.371 | 0.416 | 0.543 | 0.377 | 0.311 | 0.351 | 0.223 | 0.715 |
|  |  | **MCC** | 0.069 | 0.188 | 0.185 | 0.194 | 0.132 | 0.155 | 0.214 | 0.171 | -0.063 | 0.061 |
|  |  | **AUPRC** | 0.072 | 0.184 | 0.181 | 0.192 | 0.082 | 0.101 | 0.199 | 0.126 | 0.049 | 0.062 |
| **RWO** | **50** | **AUC** | 0.638 | 0.663 | 0.665 | 0.663 | 0.619 | 0.748 | 0.703 | 0.669 | 0.656 | 0.567 |
|  |  | **Precision** | 0.085 | 0.088 | 0.153 | 0.087 | 0.131 | 0.112 | 0.140 | 0.109 | 0.112 | 0.078 |
|  |  | **Recall** | 0.788 | 0.824 | 0.431 | 0.804 | 0.373 | 0.784 | 0.615 | 0.667 | 0.569 | 0.712 |
|  |  | **F1-score** | 0.154 | 0.159 | 0.226 | 0.157 | 0.194 | 0.196 | 0.228 | 0.187 | 0.188 | 0.140 |
|  |  | **Cohen′s Kappa** | 0.051 | 0.059 | 0.152 | 0.057 | 0.117 | 0.104 | 0.144 | 0.096 | 0.099 | 0.036 |
|  |  | **Balanced accuracy** | 0.624 | 0.645 | 0.641 | 0.638 | 0.609 | 0.698 | 0.687 | 0.663 | 0.644 | 0.587 |
|  |  | **Error rate** | 0.520 | 0.513 | 0.174 | 0.507 | 0.182 | 0.378 | 0.250 | 0.340 | 0.290 | 0.522 |
|  |  | **MCC** | 0.119 | 0.137 | 0.178 | 0.131 | 0.138 | 0.189 | 0.202 | 0.160 | 0.148 | 0.083 |
|  |  | **AUPRC** | 0.094 | 0.111 | 0.106 | 0.112 | 0.097 | 0.155 | 0.168 | 0.158 | 0.120 | 0.066 |
|  | **100** | **AUC** | 0.603 | 0.676 | 0.664 | 0.681 | 0.632 | 0.722 | 0.756 | 0.707 | 0.633 | 0.608 |
|  |  | **Precision** | 0.098 | 0.105 | 0.102 | 0.114 | 0.085 | 0.098 | 0.128 | 0.084 | 0.096 | 0.091 |
|  |  | **Recall** | 0.451 | 0.588 | 0.569 | 0.569 | 0.712 | 0.902 | 0.765 | 0.882 | 0.588 | 0.627 |
|  |  | **F1-score** | 0.161 | 0.179 | 0.174 | 0.190 | 0.153 | 0.177 | 0.220 | 0.154 | 0.166 | 0.159 |
|  |  | **Cohen′s Kappa** | 0.071 | 0.088 | 0.082 | 0.102 | 0.051 | 0.080 | 0.132 | 0.052 | 0.072 | 0.062 |
|  |  | **Balanced accuracy** | 0.596 | 0.638 | 0.629 | 0.646 | 0.613 | 0.692 | 0.720 | 0.641 | 0.622 | 0.618 |
|  |  | **Error rate** | 0.277 | 0.318 | 0.318 | 0.285 | 0.473 | 0.493 | 0.319 | 0.572 | 0.348 | 0.391 |
|  |  | **MCC** | 0.101 | 0.138 | 0.129 | 0.151 | 0.108 | 0.182 | 0.217 | 0.136 | 0.120 | 0.113 |
|  |  | **AUPRC** | 0.111 | 0.141 | 0.141 | 0.138 | 0.097 | 0.126 | 0.184 | 0.136 | 0.088 | 0.077 |
|  | **200** | **AUC** | 0.601 | 0.732 | 0.735 | 0.750 | 0.551 | 0.717 | 0.782 | 0.766 | 0.577 | 0.613 |
|  |  | **Precision** | 0.080 | 0.118 | 0.140 | 0.128 | 0.080 | 0.129 | 0.163 | 0.126 | 0.117 | 0.100 |
|  |  | **Recall** | 0.745 | 0.647 | 0.647 | 0.725 | 0.569 | 0.569 | 0.706 | 0.745 | 0.294 | 0.549 |
|  |  | **F1-score** | 0.144 | 0.199 | 0.231 | 0.218 | 0.141 | 0.210 | 0.265 | 0.216 | 0.168 | 0.170 |
|  |  | **Cohen′s Kappa** | 0.043 | 0.111 | 0.148 | 0.131 | 0.042 | 0.126 | 0.187 | 0.128 | 0.091 | 0.078 |
|  |  | **Balanced accuracy** | 0.605 | 0.672 | 0.700 | 0.709 | 0.581 | 0.664 | 0.740 | 0.711 | 0.578 | 0.621 |
|  |  | **Error rate** | 0.519 | 0.306 | 0.254 | 0.306 | 0.408 | 0.251 | 0.231 | 0.318 | 0.172 | 0.316 |
|  |  | **MCC** | 0.099 | 0.173 | 0.211 | 0.209 | 0.077 | 0.176 | 0.259 | 0.209 | 0.103 | 0.122 |
|  |  | **AUPRC** | 0.080 | 0.175 | 0.168 | 0.181 | 0.090 | 0.190 | 0.293 | 0.183 | 0.129 | 0.079 |
| **SMOTE** | **50** | **AUC** | 0.627 | 0.635 | 0.640 | 0.634 | 0.585 | 0.710 | 0.695 | 0.713 | 0.617 | 0.576 |
|  |  | **Precision** | 0.087 | 0.082 | 0.084 | 0.080 | 0.081 | 0.102 | 0.149 | 0.111 | 0.115 | 0.083 |
|  |  | **Recall** | 0.769 | 0.765 | 0.784 | 0.824 | 0.608 | 0.808 | 0.558 | 0.692 | 0.353 | 0.510 |
|  |  | **F1-score** | 0.157 | 0.149 | 0.151 | 0.146 | 0.143 | 0.181 | 0.235 | 0.191 | 0.173 | 0.143 |
|  |  | **Cohen′s Kappa** | 0.055 | 0.048 | 0.050 | 0.044 | 0.043 | 0.084 | 0.155 | 0.099 | 0.092 | 0.047 |
|  |  | **Balanced accuracy** | 0.629 | 0.616 | 0.623 | 0.617 | 0.588 | 0.678 | 0.677 | 0.670 | 0.591 | 0.580 |
|  |  | **Error rate** | 0.495 | 0.514 | 0.519 | 0.565 | 0.430 | 0.436 | 0.217 | 0.350 | 0.198 | 0.359 |
|  |  | **MCC** | 0.122 | 0.110 | 0.117 | 0.113 | 0.083 | 0.169 | 0.202 | 0.167 | 0.112 | 0.078 |
|  |  | **AUPRC** | 0.097 | 0.086 | 0.087 | 0.085 | 0.073 | 0.124 | 0.133 | 0.112 | 0.144 | 0.072 |
|  | **100** | **AUC** | 0.634 | 0.654 | 0.654 | 0.649 | 0.593 | 0.735 | 0.716 | 0.683 | 0.599 | 0.619 |
|  |  | **Precision** | 0.083 | 0.097 | 0.097 | 0.092 | 0.080 | 0.106 | 0.091 | 0.088 | 0.079 | 0.092 |
|  |  | **Recall** | 0.725 | 0.627 | 0.627 | 0.608 | 0.750 | 0.745 | 0.902 | 0.745 | 0.725 | 0.647 |
|  |  | **F1-score** | 0.149 | 0.168 | 0.168 | 0.160 | 0.144 | 0.186 | 0.165 | 0.157 | 0.143 | 0.161 |
|  |  | **Cohen′s Kappa** | 0.049 | 0.074 | 0.074 | 0.064 | 0.040 | 0.093 | 0.065 | 0.058 | 0.041 | 0.065 |
|  |  | **Balanced accuracy** | 0.613 | 0.631 | 0.632 | 0.616 | 0.600 | 0.677 | 0.669 | 0.631 | 0.600 | 0.624 |
|  |  | **Error rate** | 0.486 | 0.366 | 0.364 | 0.376 | 0.533 | 0.383 | 0.536 | 0.469 | 0.511 | 0.396 |
|  |  | **MCC** | 0.107 | 0.127 | 0.128 | 0.112 | 0.095 | 0.169 | 0.161 | 0.123 | 0.094 | 0.119 |
|  |  | **AUPRC** | 0.121 | 0.135 | 0.134 | 0.137 | 0.078 | 0.158 | 0.171 | 0.147 | 0.081 | 0.089 |
|  | **200** | **AUC** | 0.603 | 0.698 | 0.704 | 0.698 | 0.628 | 0.703 | 0.752 | 0.686 | 0.474 | 0.592 |
|  |  | **Precision** | 0.082 | 0.130 | 0.142 | 0.134 | 0.094 | 0.137 | 0.146 | 0.088 | 0.044 | 0.076 |
|  |  | **Recall** | 0.667 | 0.608 | 0.627 | 0.627 | 0.627 | 0.569 | 0.725 | 0.804 | 0.216 | 0.667 |
|  |  | **F1-score** | 0.145 | 0.215 | 0.231 | 0.221 | 0.164 | 0.221 | 0.243 | 0.158 | 0.074 | 0.136 |
|  |  | **Cohen′s Kappa** | 0.045 | 0.130 | 0.149 | 0.137 | 0.069 | 0.140 | 0.160 | 0.058 | -0.027 | 0.034 |
|  |  | **Balanced accuracy** | 0.599 | 0.677 | 0.695 | 0.687 | 0.626 | 0.673 | 0.730 | 0.640 | 0.463 | 0.580 |
|  |  | **Error rate** | 0.461 | 0.262 | 0.246 | 0.261 | 0.376 | 0.235 | 0.266 | 0.504 | 0.319 | 0.497 |
|  |  | **MCC** | 0.093 | 0.187 | 0.209 | 0.197 | 0.121 | 0.189 | 0.238 | 0.132 | -0.039 | 0.075 |
|  |  | **AUPRC** | 0.081 | 0.179 | 0.184 | 0.180 | 0.082 | 0.133 | 0.186 | 0.132 | 0.083 | 0.074 |

Table S4. Comparison of the prediction performance of methods with oversampling algorithms in the HEXA cohort using the test dataset

| **Algorithm** | **# of**  **SNP** | **Metrics** | **Ridge** | **Lasso** | **Enet** | **SCAD** | **SVM** | **RF** | **Boosting** | **Bagging** | **NB** | **KNN** |
| --- | --- | --- | --- | --- | --- | --- | --- | --- | --- | --- | --- | --- |
| **MWMOTE** | **50** | **AUC** | 0.588 | 0.612 | 0.609 | 0.612 | 0.581 | 0.657 | 0.648 | 0.639 | 0.565 | 0.563 |
|  |  | **Precision** | 0.023 | 0.025 | 0.024 | 0.025 | 0.019 | 0.030 | 0.029 | 0.026 | 0.020 | 0.020 |
|  |  | **Recall** | 0.583 | 0.647 | 0.628 | 0.641 | 0.885 | 0.532 | 0.615 | 0.583 | 0.577 | 0.622 |
|  |  | **F1-score** | 0.044 | 0.047 | 0.047 | 0.047 | 0.037 | 0.057 | 0.056 | 0.050 | 0.038 | 0.039 |
|  |  | **Cohen′s Kappa** | 0.012 | 0.016 | 0.015 | 0.016 | 0.005 | 0.026 | 0.025 | 0.018 | 0.006 | 0.007 |
|  |  | **Balanced accuracy** | 0.579 | 0.606 | 0.600 | 0.605 | 0.558 | 0.620 | 0.636 | 0.606 | 0.547 | 0.556 |
|  |  | **Error rate** | 0.426 | 0.434 | 0.428 | 0.430 | 0.758 | 0.294 | 0.345 | 0.373 | 0.482 | 0.508 |
|  |  | **MCC** | 0.041 | 0.055 | 0.052 | 0.054 | 0.035 | 0.068 | 0.073 | 0.056 | 0.024 | 0.029 |
|  |  | **AUPRC** | 0.024 | 0.033 | 0.033 | 0.033 | 0.027 | 0.027 | 0.028 | 0.026 | 0.020 | 0.020 |
|  | **100** | **AUC** | 0.554 | 0.584 | 0.584 | 0.584 | 0.574 | 0.620 | 0.624 | 0.624 | 0.515 | 0.520 |
|  |  | **Precision** | 0.020 | 0.021 | 0.022 | 0.021 | 0.023 | 0.031 | 0.023 | 0.025 | 0.018 | 0.018 |
|  |  | **Recall** | 0.737 | 0.712 | 0.737 | 0.679 | 0.481 | 0.429 | 0.737 | 0.609 | 0.538 | 0.564 |
|  |  | **F1-score** | 0.039 | 0.041 | 0.042 | 0.041 | 0.044 | 0.058 | 0.044 | 0.049 | 0.035 | 0.035 |
|  |  | **Cohen′s Kappa** | 0.007 | 0.009 | 0.010 | 0.008 | 0.012 | 0.027 | 0.012 | 0.017 | 0.003 | 0.003 |
|  |  | **Balanced accuracy** | 0.565 | 0.577 | 0.585 | 0.569 | 0.566 | 0.600 | 0.600 | 0.606 | 0.523 | 0.525 |
|  |  | **Error rate** | 0.601 | 0.553 | 0.563 | 0.537 | 0.351 | 0.234 | 0.534 | 0.396 | 0.493 | 0.513 |
|  |  | **MCC** | 0.034 | 0.040 | 0.044 | 0.036 | 0.036 | 0.061 | 0.051 | 0.056 | 0.012 | 0.013 |
|  |  | **AUPRC** | 0.019 | 0.025 | 0.025 | 0.025 | 0.020 | 0.026 | 0.025 | 0.024 | 0.017 | 0.017 |
|  | **200** | **AUC** | 0.567 | 0.593 | 0.593 | 0.597 | 0.582 | 0.639 | 0.663 | 0.673 | 0.501 | 0.532 |
|  |  | **Precision** | 0.025 | 0.026 | 0.026 | 0.021 | 0.025 | 0.025 | 0.028 | 0.028 | 0.013 | 0.023 |
|  |  | **Recall** | 0.391 | 0.417 | 0.417 | 0.782 | 0.404 | 0.718 | 0.635 | 0.654 | 0.199 | 0.218 |
|  |  | **F1-score** | 0.047 | 0.049 | 0.049 | 0.041 | 0.047 | 0.048 | 0.053 | 0.055 | 0.025 | 0.042 |
|  |  | **Cohen′s Kappa** | 0.016 | 0.018 | 0.018 | 0.009 | 0.016 | 0.016 | 0.022 | 0.023 | -0.007 | 0.012 |
|  |  | **Balanced accuracy** | 0.567 | 0.575 | 0.575 | 0.580 | 0.569 | 0.620 | 0.629 | 0.637 | 0.472 | 0.531 |
|  |  | **Error rate** | 0.263 | 0.272 | 0.272 | 0.615 | 0.271 | 0.475 | 0.376 | 0.379 | 0.264 | 0.167 |
|  |  | **MCC** | 0.039 | 0.043 | 0.043 | 0.043 | 0.040 | 0.061 | 0.068 | 0.072 | -0.016 | 0.022 |
|  |  | **AUPRC** | 0.021 | 0.030 | 0.030 | 0.031 | 0.023 | 0.027 | 0.032 | 0.031 | 0.016 | 0.019 |
| **RWO** | **50** | **AUC** | 0.590 | 0.608 | 0.608 | 0.611 | 0.610 | 0.667 | 0.702 | 0.640 | 0.589 | 0.540 |
|  |  | **Precision** | 0.020 | 0.025 | 0.025 | 0.025 | 0.000 | 0.033 | 0.044 | 0.028 | 0.021 | 0.023 |
|  |  | **Recall** | 0.808 | 0.609 | 0.609 | 0.635 | 0.000 | 0.590 | 0.513 | 0.474 | 0.647 | 0.295 |
|  |  | **F1-score** | 0.040 | 0.048 | 0.048 | 0.048 | NaN | 0.062 | 0.082 | 0.053 | 0.041 | 0.042 |
|  |  | **Cohen′s Kappa** | 0.007 | 0.016 | 0.016 | 0.017 | -0.015 | 0.031 | 0.052 | 0.022 | 0.009 | 0.011 |
|  |  | **Balanced accuracy** | 0.574 | 0.601 | 0.601 | 0.609 | 0.493 | 0.647 | 0.663 | 0.598 | 0.572 | 0.539 |
|  |  | **Error rate** | 0.652 | 0.407 | 0.407 | 0.416 | 0.030 | 0.298 | 0.193 | 0.283 | 0.502 | 0.225 |
|  |  | **MCC** | 0.040 | 0.052 | 0.053 | 0.056 | -0.015 | 0.082 | 0.106 | 0.055 | 0.037 | 0.024 |
|  |  | **AUPRC** | 0.024 | 0.033 | 0.033 | 0.033 | 0.022 | 0.038 | 0.056 | 0.038 | 0.026 | 0.020 |
|  | **100** | **AUC** | 0.556 | 0.579 | 0.579 | 0.578 | 0.587 | 0.658 | 0.702 | 0.637 | 0.535 | 0.525 |
|  |  | **Precision** | 0.020 | 0.021 | 0.021 | 0.022 | 0.028 | 0.028 | 0.040 | 0.029 | 0.020 | 0.017 |
|  |  | **Recall** | 0.731 | 0.628 | 0.628 | 0.571 | 0.436 | 0.596 | 0.558 | 0.481 | 0.712 | 1.000 |
|  |  | **F1-score** | 0.040 | 0.041 | 0.041 | 0.042 | 0.053 | 0.053 | 0.075 | 0.055 | 0.038 | 0.033 |
|  |  | **Cohen′s Kappa** | 0.008 | 0.009 | 0.009 | 0.010 | 0.023 | 0.022 | 0.046 | 0.024 | 0.006 | 0.000 |
|  |  | **Balanced accuracy** | 0.569 | 0.570 | 0.571 | 0.568 | 0.592 | 0.622 | 0.666 | 0.604 | 0.554 | 0.500 |
|  |  | **Error rate** | 0.588 | 0.485 | 0.485 | 0.434 | 0.258 | 0.353 | 0.229 | 0.277 | 0.598 | 0.983 |
|  |  | **MCC** | 0.036 | 0.036 | 0.036 | 0.035 | 0.054 | 0.065 | 0.101 | 0.060 | 0.028 | 0.000 |
|  |  | **AUPRC** | 0.019 | 0.025 | 0.025 | 0.026 | 0.022 | 0.037 | 0.054 | 0.029 | 0.018 | 0.018 |
|  | **200** | **AUC** | 0.576 | 0.603 | 0.601 | 0.604 | 0.601 | 0.643 | 0.728 | 0.675 | 0.512 | 0.530 |
|  |  | **Precision** | 0.022 | 0.031 | 0.023 | 0.021 | 0.024 | 0.024 | 0.043 | 0.027 | 0.014 | 0.023 |
|  |  | **Recall** | 0.564 | 0.327 | 0.609 | 0.788 | 0.583 | 0.615 | 0.551 | 0.686 | 0.160 | 0.212 |
|  |  | **F1-score** | 0.042 | 0.056 | 0.043 | 0.040 | 0.046 | 0.046 | 0.080 | 0.052 | 0.025 | 0.042 |
|  |  | **Cohen′s Kappa** | 0.010 | 0.027 | 0.012 | 0.008 | 0.015 | 0.015 | 0.050 | 0.020 | -0.006 | 0.012 |
|  |  | **Balanced accuracy** | 0.567 | 0.576 | 0.580 | 0.577 | 0.592 | 0.597 | 0.671 | 0.633 | 0.481 | 0.530 |
|  |  | **Error rate** | 0.431 | 0.183 | 0.448 | 0.626 | 0.399 | 0.421 | 0.212 | 0.419 | 0.209 | 0.161 |
|  |  | **MCC** | 0.035 | 0.051 | 0.041 | 0.041 | 0.048 | 0.050 | 0.107 | 0.069 | -0.012 | 0.022 |
|  |  | **AUPRC** | 0.022 | 0.028 | 0.028 | 0.028 | 0.022 | 0.040 | 0.058 | 0.042 | 0.017 | 0.019 |
| **SMOTE** | **50** | **AUC** | 0.604 | 0.629 | 0.627 | 0.629 | 0.588 | 0.662 | 0.637 | 0.630 | 0.585 | 0.545 |
|  |  | **Precision** | 0.022 | 0.024 | 0.024 | 0.024 | 0.027 | 0.026 | 0.030 | 0.029 | 0.029 | 0.021 |
|  |  | **Recall** | 0.718 | 0.679 | 0.647 | 0.679 | 0.391 | 0.731 | 0.462 | 0.468 | 0.327 | 0.468 |
|  |  | **F1-score** | 0.044 | 0.046 | 0.047 | 0.046 | 0.050 | 0.050 | 0.056 | 0.054 | 0.053 | 0.041 |
|  |  | **Cohen′s Kappa** | 0.012 | 0.014 | 0.015 | 0.014 | 0.019 | 0.019 | 0.026 | 0.023 | 0.023 | 0.009 |
|  |  | **Balanced accuracy** | 0.594 | 0.604 | 0.602 | 0.604 | 0.574 | 0.634 | 0.604 | 0.599 | 0.570 | 0.551 |
|  |  | **Error rate** | 0.526 | 0.469 | 0.441 | 0.470 | 0.249 | 0.460 | 0.258 | 0.274 | 0.195 | 0.368 |
|  |  | **MCC** | 0.048 | 0.053 | 0.053 | 0.053 | 0.044 | 0.069 | 0.061 | 0.057 | 0.046 | 0.027 |
|  |  | **AUPRC** | 0.032 | 0.038 | 0.038 | 0.038 | 0.022 | 0.029 | 0.030 | 0.027 | 0.023 | 0.019 |
|  | **100** | **AUC** | 0.572 | 0.601 | 0.602 | 0.600 | 0.570 | 0.639 | 0.649 | 0.621 | 0.561 | 0.546 |
|  |  | **Precision** | 0.020 | 0.023 | 0.023 | 0.022 | 0.020 | 0.024 | 0.035 | 0.023 | 0.024 | 0.021 |
|  |  | **Recall** | 0.756 | 0.590 | 0.583 | 0.609 | 0.654 | 0.712 | 0.468 | 0.558 | 0.481 | 0.468 |
|  |  | **F1-score** | 0.039 | 0.045 | 0.044 | 0.043 | 0.039 | 0.047 | 0.066 | 0.044 | 0.046 | 0.040 |
|  |  | **Cohen′s Kappa** | 0.007 | 0.013 | 0.012 | 0.011 | 0.007 | 0.015 | 0.036 | 0.012 | 0.014 | 0.008 |
|  |  | **Balanced accuracy** | 0.567 | 0.585 | 0.581 | 0.579 | 0.559 | 0.612 | 0.626 | 0.577 | 0.575 | 0.548 |
|  |  | **Error rate** | 0.616 | 0.420 | 0.422 | 0.451 | 0.532 | 0.485 | 0.221 | 0.405 | 0.334 | 0.375 |
|  |  | **MCC** | 0.035 | 0.044 | 0.042 | 0.040 | 0.030 | 0.057 | 0.078 | 0.040 | 0.041 | 0.025 |
|  |  | **AUPRC** | 0.023 | 0.028 | 0.028 | 0.028 | 0.022 | 0.029 | 0.032 | 0.026 | 0.023 | 0.019 |
|  | **200** | **AUC** | 0.594 | 0.602 | 0.602 | 0.605 | 0.593 | 0.677 | 0.665 | 0.642 | 0.585 | 0.571 |
|  |  | **Precision** | 0.021 | 0.023 | 0.023 | 0.023 | 0.022 | 0.028 | 0.029 | 0.022 | 0.021 | 0.023 |
|  |  | **Recall** | 0.840 | 0.551 | 0.551 | 0.596 | 0.724 | 0.724 | 0.596 | 0.788 | 0.622 | 0.481 |
|  |  | **F1-score** | 0.041 | 0.044 | 0.044 | 0.044 | 0.042 | 0.053 | 0.055 | 0.043 | 0.041 | 0.044 |
|  |  | **Cohen′s Kappa** | 0.009 | 0.012 | 0.012 | 0.012 | 0.010 | 0.022 | 0.024 | 0.011 | 0.009 | 0.013 |
|  |  | **Balanced accuracy** | 0.589 | 0.575 | 0.575 | 0.580 | 0.584 | 0.645 | 0.628 | 0.601 | 0.570 | 0.568 |
|  |  | **Error rate** | 0.653 | 0.403 | 0.402 | 0.435 | 0.551 | 0.432 | 0.342 | 0.581 | 0.480 | 0.348 |
|  |  | **MCC** | 0.048 | 0.039 | 0.039 | 0.041 | 0.044 | 0.075 | 0.069 | 0.052 | 0.036 | 0.036 |
|  |  | **AUPRC** | 0.021 | 0.037 | 0.032 | 0.036 | 0.023 | 0.030 | 0.036 | 0.027 | 0.022 | 0.022 |

Table S5. List of prioritized putative single nucleotide polymorphisms

| **CHR^a^** | **POS^b^** | **rsID** | **Annotation** | **Gene** | **CADD** | **DANN** | **References** |
| --- | --- | --- | --- | --- | --- | --- | --- |
| **CAVAS cohort** | | | | | | | |
| 6 | 20726035 | rs6914868 | Upstream | *RP3-348I23.3* | 10.86 | 0.889 | [69] |
| 15 | 40542199 | rs2277562 | Upstream | *PAK6* | 13.35 | 0.798 | [75, 85] |
| 17 | 46696416 | rs3809775 | Upstream | *HOXB8* | 11.31 | 0.636 | [65] |
| **KARE cohort** | | | | | | | |
| 4 | 102064291 | rs2850997 | Intron | *PPP3CA* | 12.26 | 0.911 | [64, 83] |
| 4 | 102066380 | rs2695207 | Intron | *PPP3CA* | 10.92 | 0.919 | [64, 83] |
| 4 | 131534121 | rs6821303 | Intergenic | *GAPDHP56-RP11-401I19.1* | 25.9 | 0.619 | [74, 77, 84] |
| 4 | 151492574 | rs77274548 | Intron | *LRBA* | 13.27 | 0.702 | [68, 78] |
| 7 | 81567479 | rs141890899 | Intergenic | *AC006145.1-CACNA2D1* | 13.19 | 0.981 | [79] |
| **HEXA cohort** | | | | | | | |
| 2 | 228092812 | rs73996240 | Intron | *COL4A3* | 11.73 | 0.833 | [67] |
| 4 | 137728814 | rs62313713 | Downstream | *RP11-138I17.1* | 20.3 | 0.85 | [74, 77, 84] |
| 7 | 141845864 | rs13224443 | Downstream | *RP11-1220K2.2* | 15.34 | 0.908 | [74, 77, 84] |
| 8 | 22437141 | rs11786352 | Upstream | *PDLIM2* | 11.66 | 0.919 | [80] |
| 12 | 96444697 | rs2660851 | Intergenic | *LTA4H-RP11-256L6.4* | 13.53 | 0.934 | [70, 74, 76, 77, 81, 82, 84] |
| 13 | 74318497 | rs1535802 | Intron | *KLF12* | 13.79 | 0.72 | [66, 72] |
| 14 | 64476680 | rs7146588 | Splice & Intron | *SYNE2* | 12.67 | 0.616 | [71, 73] |

^a^ Chromosome; ^b^ Base-pair position; Cutoff: CADD ≥ 10 and DANN ≥ 0.6.

**Reference for Additional file 1**

64. Lin C-C, Law BF, Hettick JM: **Acute 4, 4′-Methylene Diphenyl Diisocyanate Exposure-Mediated Downregulation of miR-206-3p and miR-381-3p Activates Inducible Nitric Oxide Synthase Transcription by Targeting Calcineurin/NFAT Signaling in Macrophages**. *Toxicological Sciences* 2020, **173**(1):100-113.

65. Li L-J, Gao L-B, Lv M-L, Dong W, Su X-W, Liang W-B, Zhang L: **Association between SNPs in pre-miRNA and risk of chronic obstructive pulmonary disease**. *Clinical biochemistry* 2011, **44**(10-11):813-816.

66. Akat A, Yilmaz Semerci S, Ugurel OM, Erdemir A, Danhaive O, Cetinkaya M, Turgut-Balik D: **Bronchopulmonary dysplasia and wnt pathway-associated single nucleotide polymorphisms**. *Pediatric Research* 2021:1-11.

67. Nemani SSP, Vermeulen CJ, Pech M, Faiz A, Oliver BGG, van den Berge M, Burgess JK, Kopp MV, Weckmann M: **COL4A3 expression in asthmatic epithelium depends on intronic methylation and ZNF263 binding**. *ERJ open research* 2021, **7**(2).

68. Lopez-Herrera G, Tampella G, Pan-Hammarström Q, Herholz P, Trujillo-Vargas CM, Phadwal K, Simon AK, Moutschen M, Etzioni A, Mory A: **Deleterious mutations in LRBA are associated with a syndrome of immune deficiency and autoimmunity**. *The American Journal of Human Genetics* 2012, **90**(6):986-1001.

69. Yang Y, Haitchi HM, Cakebread J, Sammut D, Harvey A, Powell RM, Holloway JW, Howarth P, Holgate ST, Davies DE: **Epigenetic mechanisms silence a disintegrin and metalloprotease 33 expression in bronchial epithelial cells**. *Journal of allergy and clinical immunology* 2008, **121**(6):1393-1399. e1314.

70. Szul T, Castaldi P, Cho MH, Blalock JE, Gaggar A: **Genetic regulation of expression of leukotriene A4 hydrolase**. *ERJ Open Research* 2016, **2**(1).

71. Imboden M, Bouzigon E, Curjuric I, Ramasamy A, Kumar A, Hancock DB, Wilk JB, Vonk JM, Thun GA, Siroux V *et al*: **Genome-wide association study of lung function decline in adults with and without asthma**. *Journal of Allergy and Clinical Immunology* 2012, **129**(5):1218-1228.

72. Sin S, Choi H-M, Lim J, Kim J, Bak SH, Choi SS, Park J, Lee JH, Oh Y-M, Lee MK: **A genome-wide association study of quantitative computed tomographic emphysema in Korean populations**. *Scientific reports* 2021, **11**(1):1-10.

73. Bérubé J-C, Gaudreault N, Lavoie-Charland E, Sbarra L, Henry C, Madore A-M, Paré PD, van den Berge M, Nickle D, Laviolette M: **Identification of susceptibility genes of adult asthma in French Canadian women**. *Canadian respiratory journal* 2016, **2016**.

74. Gál Z, Gézsi A, Semsei ÁF, Nagy A, Sultész M, Csoma Z, Tamási L, Gálffy G, Szalai C: **Investigation of circulating lncRNAs as potential biomarkers in chronic respiratory diseases**. *Journal of translational medicine* 2020, **18**(1):1-15.

75. Suzuki M, Cole JJ, Konno S, Makita H, Kimura H, Nishimura M, Maciewicz RA: **Large‐scale plasma proteomics can reveal distinct endotypes in chronic obstructive pulmonary disease and severe asthma**. *Clinical and translational allergy* 2021, **11**(10):e12091.

76. Tulah AS, Beghé B, Barton SJ, Holloway JW, Sayers I: **Leukotriene B4 receptor locus gene characterisation and association studies in asthma**. *BMC medical genetics* 2012, **13**(1):1-11.

77. Li C, Liu H, Zhang J, Zhang J, Dai L, Zhao Z, Fang L, Liu L, Shu J, Feng J: **LncRNA BMF-AS1 Exerts Anti-Apoptosis Function in COPD by Regulating BMF Expression**. *Age (Mean±SD, year)* 2020, **71**(5):65-64.

78. Alangari A, Alsultan A, Adly N, Massaad MJ, Kiani IS, Aljebreen A, Raddaoui E, Almomen A-K, Al-Muhsen S, Geha RS: **LPS-responsive beige-like anchor (LRBA) gene mutation in a family with inflammatory bowel disease and combined immunodeficiency**. *Journal of Allergy and Clinical Immunology* 2012, **130**(2):481-488. e482.

79. Michalik M, Samet A, Dmowska-Koroblewska A, Podbielska-Kubera A, Waszczuk-Jankowska M, Struck-Lewicka W, Markuszewski MJ: **An overview of the application of systems biology in an understanding of chronic rhinosinusitis (CRS) development**. *Journal of personalized medicine* 2020, **10**(4):245.

80. Tanaka T, Yamamoto Y, Muromoto R, Ikeda O, Sekine Y, Grusby MJ, Kaisho T, Matsuda T: **PDLIM2 inhibits T helper 17 cell development and granulomatous inflammation through degradation of STAT3**. *Science signaling* 2011, **4**(202):ra85-ra85.

81. Via M, De Giacomo A, Corvol H, Eng C, Seibold MA, Gillett C, Galanter J, Sen S, Tcheurekdjian H, Chapela R: **The role of LTA4H and ALOX5AP genes in the risk for asthma in Latinos**. *Clinical & Experimental Allergy* 2010, **40**(4):582-589.

82. Holloway J, Barton S, Holgate S, Rose‐Zerilli M, Sayers I: **The role of LTA4H and ALOX5AP polymorphism in asthma and allergy susceptibility**. *Allergy* 2008, **63**(8):1046-1053.

83. Kim J, Kim DY, Heo H-R, Choi SS, Hong S-H, Kim WJ: **Role of miRNA-181a-2-3p in cadmium-induced inflammatory responses of human bronchial epithelial cells**. *Journal of thoracic disease* 2019, **11**(7):3055.

84. Perry MM, Tsitsiou E, Austin PJ, Lindsay MA, Gibeon DS, Adcock IM, Chung KF: **Role of non-coding RNAs in maintaining primary airway smooth muscle cells**. *Respiratory research* 2014, **15**(1):1-12.

85. Hayden LP, Cho MH, McDonald M-LN, Crapo JD, Beaty TH, Silverman EK, Hersh CP: **Susceptibility to childhood pneumonia: a genome-wide analysis**. *American journal of respiratory cell and molecular biology* 2017, **56**(1):20-28.
